# Therapeutic Effects of Catechins in Less Common Neurological and Neurodegenerative Disorders

**DOI:** 10.3390/nu13072232

**Published:** 2021-06-29

**Authors:** Giorgia Sebastiani, Laura Almeida-Toledano, Mariona Serra-Delgado, Elisabet Navarro-Tapia, Sebastian Sailer, Olga Valverde, Oscar Garcia-Algar, Vicente Andreu-Fernández

**Affiliations:** 1 Department of Neonatology, Hospital Clínic-Maternitat, ICGON, BCNatal, 08028 Barcelona, Spain; gsebasti@clinic.cat (G.S.); sebastiansailer34@gmail.com (S.S.); ogarciaa@clinic.cat (O.G.-A.); 2 Institut de Recerca Sant Joan de Déu, 08950 Esplugues de Llobregat, Spain; lalmeida@sjdhospitalbarcelona.org (L.A.-T.); mserrad@sjdhospitalbarcelona.or (M.S.-D.); 3 BCNatal, Fetal Medicine Research Center (Hospital Sant Joan de Déu and Hospital Clínic), University of Barcelona, 08950 Barcelona, Spain; 4 Grup de Recerca Infancia i Entorn (GRIE), Institut d’Investigacions Biomèdiques August Pi i Sunyer (IDIBAPS), 08036 Barcelona, Spain; elisabetnavarrotapia@gmail.com; 5 Department of Neonatology, Kepler University Hospital, Johannes Kepler University, Faculty of Medicine, Krankenhausstrasse 26–30, 4020 Linz, Austria; 6 Neurobiology of Behaviour Research Group (GReNeC-NeuroBio), Department of Experimental and Health Sciences, Universitat Pompeu Fabra, 08003 Barcelona, Spain; olga.valverde@upf.edu; 7 Neuroscience Research Programme, IMIM-Hospital del Mar Research Institute, 08003 Barcelona, Spain; 8 Department of Nutrition and Health, Valencian International University (VIU), 46002 Valencia, Spain

**Keywords:** catechins, epigallocatechin-3-gallate, antioxidant, neurodegenerative disorders, neurological disorders, multiple sclerosis, fetal alcohol spectrum disorders, Down syndrome, age-related cognitive decline

## Abstract

In recent years, neurological and neurodegenerative disorders research has focused on altered molecular mechanisms in search of potential pharmacological targets, e.g., imbalances in mechanisms of response to oxidative stress, inflammation, apoptosis, autophagy, proliferation, differentiation, migration, and neuronal plasticity, which occur in less common neurological and neurodegenerative pathologies (Huntington disease, multiple sclerosis, fetal alcohol spectrum disorders, and Down syndrome). Here, we assess the effects of different catechins (particularly of epigalocatechin-3-gallate, EGCG) on these disorders, as well as their use in attenuating age-related cognitive decline in healthy individuals. Antioxidant and free radical scavenging properties of EGCG -due to their phenolic hydroxyl groups-, as well as its immunomodulatory, neuritogenic, and autophagic characteristics, makes this catechin a promising tool against neuroinflammation and microglia activation, common in these pathologies. Although EGCG promotes the inhibition of protein aggregation in experimental Huntington disease studies and improves the clinical severity in multiple sclerosis in animal models, its efficacy in humans remains controversial. EGCG may normalize DYRK1A (involved in neural plasticity) overproduction in Down syndrome, improving behavioral and neural phenotypes. In neurological pathologies caused by environmental agents, such as FASD, EGCG enhances antioxidant defense and regulates placental angiogenesis and neurodevelopmental processes. As demonstrated in animal models, catechins attenuate age-related cognitive decline, which results in improvements in long-term outcomes and working memory, reduction of hippocampal neuroinflammation, and enhancement of neuronal plasticity; however, further studies are needed. Catechins are valuable compounds for treating and preventing certain neurodegenerative and neurological diseases of genetic and environmental origin. However, the use of different doses of green tea extracts and EGCG makes it difficult to reach consistent conclusions for different populations.

## 1. Introduction

Catechins are polyphenolic flavonoids derived from catechu, which is the tannic juice or boiled extract of *Acacia catechu* L. Green tea, one of the most consumed beverages worldwide, obtained from the buds and leaves of the plant *Camellia sinensis*, is a well-known source of catechins. Moreover, catechins are found in a variety of foods and herbs including wine, apples, persimmons, cocoa, grapes, berries, and cocoa-based products. Due to numerous hydroxyl groups, catechins have powerful antioxidant and metal-chelating properties, which have been confirmed in in-vitro and clinical studies [1,2,3].

In recent years, (-)-epigallocatechin-3-gallate (EGCG) and (-)-epicatechin-3-gallate (ECG) have been the subject of multiple studies due to their anti-cancer, anti-obesity, anti-diabetic, anti-cardiovascular, anti-infectious, or hepatoprotective effects [4]. Some works have focused on the use of catechins on neurodegenerative disorders, e.g., Alzheimer’s disease and Parkinson’s disease [5,6,7,8]. The present review is centered on the potential application of these antioxidants as therapeutic compounds in less-common neurological disorders, neurodegenerative disorders, and age-related cognitive decline.

Recently, catechins have attracted attention in the field of neurodegenerative disorders like Huntington disease (HD) (estimated prevalence of 2.7–5.7/100,000) and multiple sclerosis (MS), the most frequent cause of permanent non-traumatic disability in young adults, with high-risk areas in Europe, Canada, United States, New Zealand, and Australia (prevalence > 60/100,000) [9,10,11,12]. We further review the effect of catechins on Down syndrome (DS) and Fetal Alcohol Spectrum Disorders (FASD). DS is the most common chromosome abnormality among newborns (trisomy 21), with an estimated prevalence of 23/10,000 births [13,14], while FASD is caused by the toxic and teratogenic effects of prenatal alcohol consumption, with global and European prevalence of 7.7/1000 and 19.8/1000, respectively [15,16]. Recent evidence shows a common cellular and molecular origin disrupted by trisomy and alcohol intake, leading to craniofacial and neurocognitive phenotypes of DS and FASD [17].

This article provides an overview of current knowledge regarding catechins, differences between polyphenol classes, their chemical structures and features. We also discuss the neuroprotective molecular mechanisms of catechins, clinical implementation in less-common neurological and neurodegenerative disorders, and their use in the healthy population. The pathophysiological aspects of each disease, their clinical manifestations, and current treatments are addressed in the corresponding sections.

## 2. Materials and Methods

**Figure 1:**
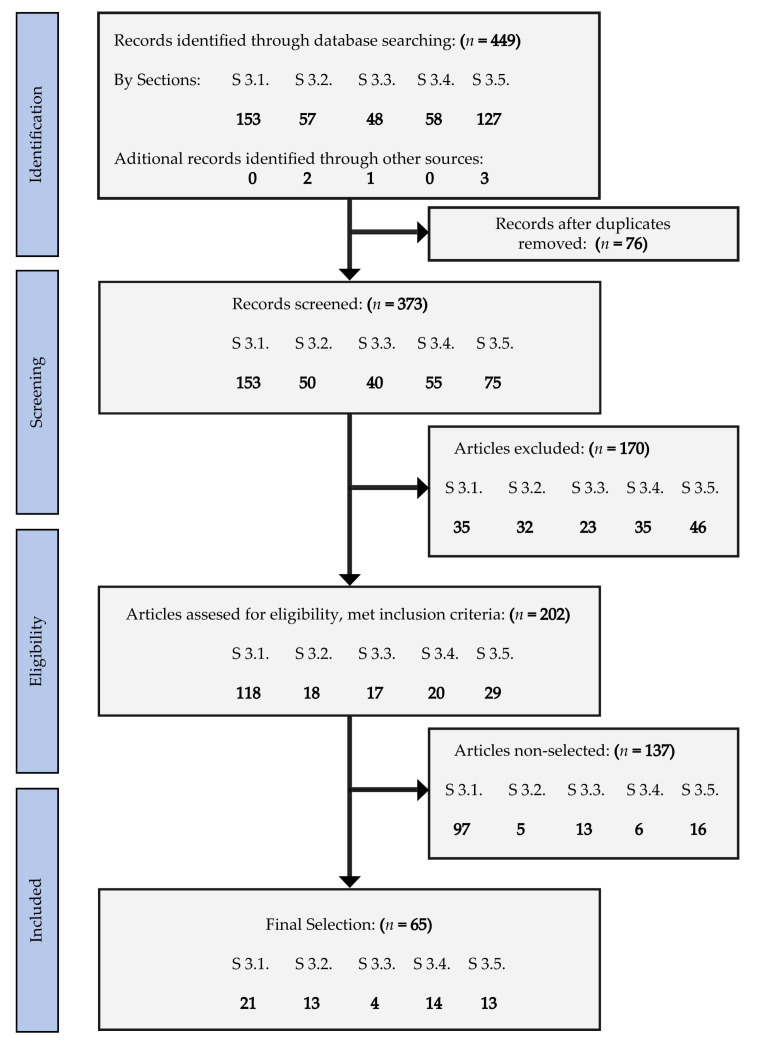
Methodological flowchart following the Preferred Reporting Items for Systematic Reviews and Meta-Analyses (PRISMA) guidelines.

We followed the Preferred Reporting Items for Systematic Reviews and Meta-Analyses (PRISMA) statement [18,19] to conduct this review, including definition of the research question and objectives, bibliographic search and data collection, evaluation, synthesis and comparison, critical appraisal of the analyzed scientific papers, as well as presentation of the main findings and conclusions, showing the strengths and weaknesses of the studies (Figure 1). The objective of the present study was to assess the therapeutic effects of different catechins on neurological alterations and neurodegenerative diseases. We decided not to perform a meta-analysis because of the different experimental designs and outcome variables observed by the clinical particularities and the differential effects of catechins on these pathologies, and the scarce number of catechins-based studies found for some of these pathologies that might lead to important bias in the statistical results.

Due to the large number of studies on the effects of EGCG treatment on Alzheimer’s disease and Parkinson’s disease, we excluded these pathologies from our bibliographic search and focused on the use of catechins in the management of other neurological and neurodegenerative disorders, i.e., multiple sclerosis, Huntington disease, Down syndrome, as well as fetal alcohol spectrum disorders. We also assessed the neurocognitive effects of catechin intake to mitigate age-related cognitive decline in healthy populations. PubMed (MeSH), Web of science, Cochrane Central Register of Controlled Trials, and Scopus were the consulted electronic databases using the following descriptors (as MeSH terms or not) with the Boolean operators (AND/OR) in multiple combinations (see Supplementary Materials: Methodology) Section 3.1. “((catechins NOT polyphenols) AND (antioxidant OR anti-inflammatory) AND (neurodegenerative)); ((catechins OR epicagallotechin-3-gallate) AND (neuritogenesis)); ((autophagy) AND (catechins OR epigallocatechin-3-gallate) AND (neuron OR neurodegenerative)); ((catechins OR epigallocatechin-3-gallate) AND (DYRK1A))”; Section 3.2. “((catechins OR epigallocatechin-3-gallate) AND Huntington disease); ((catechins OR epigallocatechin-3-gallate) AND Multiple sclerosis)”; Section 3.3. “((Catechins OR epigallocatechin-3-gallate OR Green tea) AND (Fetal Alcohol Spectrum Disorders OR prenatal Alcohol-Related Disorders))”; Section 3.4. ““((Catechins OR epigallocatechin-3-gallate OR Green tea) AND Down syndrome)”; Section 3.5. “((Catechins OR epigallocatechin-3-gallate OR Green tea) AND (neurocognitive response OR age-related cognitive decline) ((Catechins OR epigallocatechin-3-gallate OR Green tea) AND health AND (neurology OR mental OR cognitive))”.

Inclusion criteria were papers written in English published between 1 January 2010 and 31 January 2021, presence of the selected terms in the title or as keywords, original research performed in humans, as well as in murine models due to the few published studies on the use of catechins for the treatment of MS and FASD. Type of selected experimental designs were classical articles, clinical studies, clinical trials, comparative studies, case–control studies, longitudinal cohorts, cross-sectional, and case report studies with minimum sample size of 10 participants. Exclusion criteria were non-systematic reviews, interventions using other natural compounds or drugs that may interfere with the specific effects of catechins on these pathologies, presence of other related diseases, and papers with results showing neurological, mood, or CNS outputs following catechin treatment.

Original manuscripts were selected by screening titles and abstracts, and by creating a reference list of papers for the pathologies of interest for this review. The following information was evaluated from each study: first author, experimental design, number of participants, control groups, main outcomes/findings, conclusions, and strengths and limitations (including biases). The eligibility criteria was based on the PICOS elements whenever possible. Population: men or women diagnosed with some of the neurological or neurodegenerative diseases included in this study (MS, HD, DS, and FASD, as well as age-related cognitive decline in healthy populations). Intervention: catechin administration at any dose. Comparison: placebo if applicable. Outcomes: primary outcome was the neurocognitive response of patients or animal models, changes in levels or expression of molecular biomarkers associated with diverse neurological functions. All authors critically assessed the selected studies based on the inclusion criteria, analyzing the methodology and key results.

Heterogeneous results were obtained following the PRISMA guidelines. This is explained by the need to include human and animal model studies, variability of the analyzed populations (patients with different age and neurological or neurodegenerative disorders), small sample size in many of the studies, and low number of randomized trials in which catechins are evaluated as potential agents for pharmacological treatment of these neurological pathologies.

Quality of evidence was assessed using the Grading of Recommendation, Assessment, Development, and Evaluation (GRADE) approach with four levels of quality: high, moderate, low, and very low [20,21]. Quality of evidence was judged ( Supplementary Tables S1–S4) by all authors, focusing on experimental designs, number of subjects, risk of bias, inconsistencies, indirectness, imprecisions, and observed relative or absolute effects. Bias was evaluated by limitations in the design of the study and execution. Inconsistency was defined as the unexplained heterogeneity of results. Indirectness was assessed by the absence of direct evidence (differences in population of study or intervention, surrogate outcomes and different comparisons). Imprecision was evaluated by the sample size or the number of events (wide confidence interval). The quality of evidence was considered as the strength of certainty of the study’s design, the effects, the relevance of outcomes, the type of interventions, and results. The quality of evidence determines if the magnitude of the effect described is correct. Authors took into account that Randomized Clinical Trial (RCT) starts with a high rating and observational studies with low rating. Rating may be changed downward because of study limitations, imprecision, inconsistency of results, indirectness of evidence, and publication bias, while rating may be modified upward for large magnitude effects, dose response, and confounders which probably minimize the effect. Inconsistency refers to an unclear heterogeneity of results. The authors followed these guidelines reviewing all the information to elaborate a final decision about which outcomes are important, hence indicating the rating of quality of evidence.

## 3. Results

Four hundred and forty-nine articles on the pathologies of interest for this review were identified, distributed as follows: Section 3.1 (153), Section 3.2 (57), Section 3.3 (48), Section 3.4 (58), and Section 3.5 (127). Seventy-six duplicates were found and eliminated, leaving 373 articles, from which 170 met the exclusion criteria and were removed. Full-text screening was performed for the remaining 202 articles and 65 met the eligibility criteria (Figure 1): Section 3.1 (21), Section 3.2 (13), Section 3.3 (4), Section 3.4 (14), and Section 3.5 (13).

**Figure 2:**
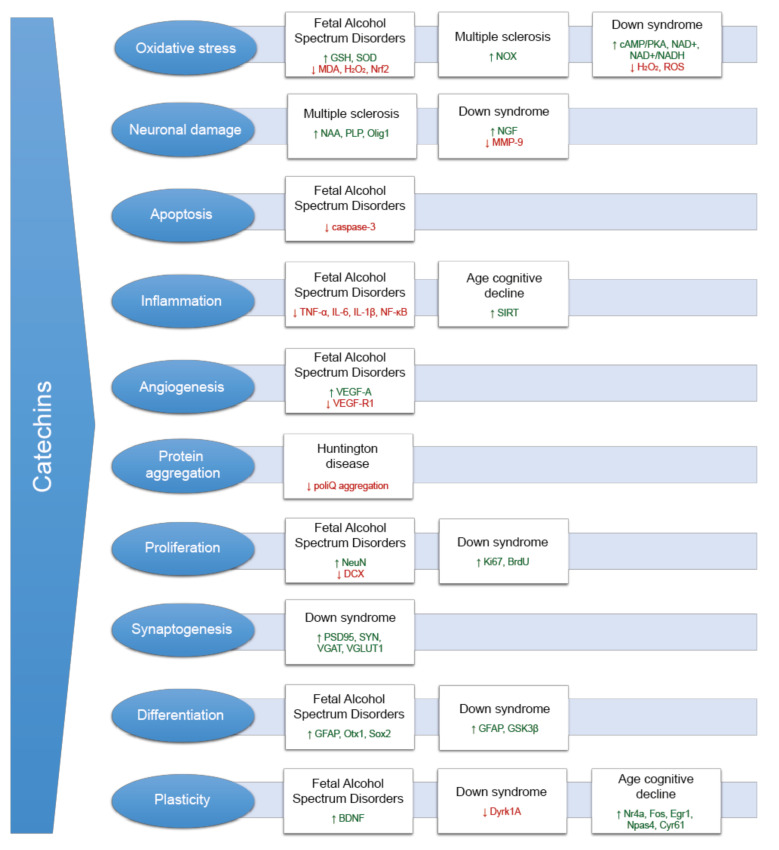
Molecular mechanisms of catechin action and changes in related biomarkers in different neurological disorders. Abbreviations: GSH: glutathione; SOD: superoxide dismutase; MDA: malondialdehyde; H_2_O_2_: hydrogen peroxide; Nrf2: nuclear factor erythroid 2-related factor 2; NOX: nitrogen oxides; cAMP/PKA: cyclic adenosine monophosphate/protein kinase A; NAD: nicotinamide adenine dinucleotide; ROS: reactive oxygen species; NAA: N-acetyl aspartate; PLP: proteolipid protein; Olig 1: oligodendrocyte transcription factor 1; NGF: nerve growth factor; MMP-9: metalloproteinase 9; TNF: tumor necrosis factor; IL: interleukin; NFκB: nuclear factor kappa-light-chain-enhancer of activated B cells; SIRT: sirtuin 1; VEGF-A: vascular endothelial growth factor A; VEGF-R1: vascular endothelial growth factor receptor 1; NeuN: neuronal nuclei; DXC: doublecortin; BrdU: 5-bromo-2′-deoxyuridine; PSD95: postsynaptic density protein 95; SYN: synaptophysin; VGAT: vesicular GABA transporter; VGLUT1: vesicular glutamate transporter 1; GFAP: glial fibrillary acidic protein; Otx1: orthodenticle homolog 1 [Drosophila]; Sox2: sex determining region Y [Sry]-related high mobility group box2; GSK3β: glycogen synthase kinase 3 beta; BDNF: brain-derived neurotrophic factor; DYRK1A: dual-specificity tyrosine phosphorylation regulated kinase 1A.

Figure 2 shows the molecular mechanisms and biomarkers altered in uncommon neurological and neurodegenerative disorders modulated by catechin treatments, described in the sections below.

### 3.1. Molecular Mechanisms Associated to Catechin Use in Neurodegenerative Disorders

Flavonoids are low molecular weight substances produced by almost all vascular plants. They have several groups, including flavan-3-ols, flavonols, flavones, isoflavones, flavanones, and anthocyanins. All are phenolic compounds with a 15-carbon skeleton arranged in three rings: two six-carbon benzene rings (A and B) connected by a pyran or pyrone heterocyclic ring C. Flavan-3-ols, often called catechins, have a 2-phenyl-3,4-dihydro-2H-chromen-3-ol as the functional moiety or backbone; some representatives of this group are catechin, epicatechin (EC), gallocatechin, ECG, and EGCG [22]. EGCG accounts for around 60% of catechins in green tea [4]; it includes a benzenediol ring, a pyrogallol ring, a tetrahydropyran moiety, and a galloyl group. The biological action of the molecule will be determined by its chemical structure. For example, the number of hydroxyl groups and their position in the molecule will affect its interaction with the biological matrix [23]. EGCG has been reported to have greater antioxidant properties than EGC or EC [24] due to a higher number of hydroxyls. Moreover, EGCG presents two structures (the ortho-3′,4′-dihydroxy moiety and the 4-keto, 3-hydroxyl or 4-keto and 5-hydroxyl moiety) that allow chelating metal ions, neutralizing their activity [25]. Therefore, catechin use, and particularly EGCG, may provide beneficial effects on certain neurodegenerative disorders.

#### 3.1.1. Antioxidant and Anti-Inflammatory Activity

The best-known biological activities of catechins are their antioxidant and free radical scavenging properties. Exacerbated levels of reactive oxygen species (ROS) damage cellular structures such as lipids, proteins, DNA, and nucleic acids; the accumulation of dysfunctional mitochondria in neuronal cells is associated with neurodegenerative disorders like Alzheimer’s disease (AD) [26]. Catechins inhibit lipid peroxidation [27]; phenolic hydroxyl groups are responsible of their antioxidant properties, which act as free radical scavengers that stop the cycle of new radical generation and regulate the synthesis of proteins related to the maintenance of redox balance, e.g., superoxide dismutase (SOD), catalase (CAT), glutathione (GSH), and NADPH [3]. Catechins, as EGCG, upregulate the activity of nuclear factor erythroid 2-related factor 2 (NRF2) by phosphorylating the p38MAPK and ERk1/2 signaling pathways [28]. In a rat neuronal cell line, EGCG significantly increased the levels of Nrf2, a regulator of cellular resistance to oxidants that is reduced in the hippocampus of AD patients [29], and enhances HO-1 expression, which regulates cell adaptation to oxidative stress protecting against cell death [30].

Catechins have immunomodulatory properties that regulate neuroinflammation, characterized by microglial activation. Moreover, they significantly contribute to progression of neurodegenerative diseases, such as AD, Parkinson’s disease (PD), HD, and amyotrophic lateral sclerosis [31]. Co-cultures of lipopolysaccharide (LPS) and the main catechins in green tea have shown to significantly decrease tumor necrosis factor (TNF-α) and interleukin (IL)-6 compared with the control group in human neutrophils. Catechins suppress protein expression of the toll-like receptor 4 (TLR4) and nuclear factor kappa-light-chain-enhancer of activated B cells (NFκB), and induce antioxidant enzyme activities and Nrf2 mRNA levels [32]. In in-vitro neuroinflammation models, EGCG improves the production of nitric oxide and TNF-α following LPS, decreases the pro-inflammatory effect produced by LPS in a Parkinsonian syndrome rat model, and restores motor impairment [33]. EGCG intake for four months reduced β-amyloid (Aβ) plaques in the hippocampus and alleviated microglia activation in an APP/PS1 transgenic mice model. The authors also observed lower levels of IL-1β and higher levels of anti-inflammatory cytokines IL-10 and IL-13 [34]. Overall, these results suggest that catechins exert their neuroprotective effects by modulating microglia activation and decreasing the production of inflammatory mediators via the NF-κB, Nrf-2, and TLR4/NF-κB pathways. In addition, catechins have iron-chelating properties, thus, they prevent the generation of free radicals by inhibiting the accumulation of iron in neurons and microglia, promoted by changes in the divalent metal transporter 1 (DMT1) and ferroportin 1 (FPN1) [35]. Iron causes aggregation of Aβ to form toxic aggregates, which accumulate in the brain in neurodegenerative diseases [36]. Thus, EGCG may influence Aβ levels, either via translational inhibition of amyloid precursor protein (APP) due to a decrease in labile Fe2+, destabilization of Aβ plaques, or avoidance of aggregation of hyperphosphorylated tau (PHFτ) [8].

#### 3.1.2. Neuritogenic and Autophagic Activity

EGCG and its metabolites have showed neuritogenic activity in human neuroblastoma SH-SY5Y cells [37]. Moreover, extremely low concentrations of unfractionated green tea polyphenols and low concentrations (<0.5 μM) of EGCG, potentiated the neuritogenic ability of the brain-derived neurotrophic factor (BDNF) in PC12(TrkB) cells [38,39]. However, in-vivo studies are needed to corroborate these results.

Aging is associated with dysfunction of the autophagy activity, which protects neuronal cells by recycling obsolete cellular constituents and removing protein aggregates, contributors to neurodegenerative disorders, such as PD [40,41]. Catechins modulate autophagy through various mechanisms, including the transcription factor EB (TFEB), the mechanistic target of rapamycin (mTOR) and 5′ AMP-activated protein kinase (AMPK) [42,43]. Khalil et al., found that EGCG restored the expression of Atg5 and LC3, important autophagy genes, via reduction of DNA methyltransferase 2 (DNMT2) gene expression in vivo and in vitro. [44]. Interestingly, learning and memory deficits in rats under chronic unpredictable mild stress (CUMS), were rescued with chronic EGCG treatment, attenuating neuronal damage in the hippocampal CA1 region. This amelioration was due to the restoration of autophagic flux, decrease of apoptotic cells, and reduction of soluble and insoluble Aβ1–42 levels in the hippocampal CA1 region of stressed rats [45]. Catechins increase the amount of available Beclin-1 [46], a protein that regulates endocytosis and autophagy. Catechins also exert neuroprotective effects through the protein kinase C (PKC) signaling cascade [47], reducing the levels of apoptotic markers such as Bad, Bax, caspase-3, and poly(ADP-ribose) polymerase (PARP) [48].

#### 3.1.3. Inhibition of Dual-Specificity Tyrosine Phosphorylation Regulated Kinase 1A

Preclinical and clinical trials have shown that EGCG is an inhibitor of the serine/threonine kinase known as inhibition of dual-specificity tyrosine phosphorylation regulated kinase 1A (DYRK1A). DYRK1A is located on 21q22.2 of the human chromosome 21 and is considered a major contributor of cognitive dysfunctions in DS due to its role in neurogenesis, neuronal differentiation, cell death, and synaptic plasticity [49]. A nutritional supplement enriched with EGCG (94% EGCG of total catechins) showed a normalization of relevant plasma and neuronal biomarkers in mice overexpressing DYRK1A, such as BDNF and NFkB. Moreover, EGCG was able to cross the blood-brain barrier, diffusing in the brain, and has demonstrated to be safe on liver and heart function [50].

Efforts invested in recent years to shed light on the molecular mechanisms of catechins, support their potential use within therapeutic strategies for the prevention and management of neurodegenerative diseases.

### 3.2. Catechin Use in Neurodegenerative Disorders

Neurodegeneration resulting from less common neurological disorders, such as HD and MS, is characterized by different pathophysiological mechanisms. Hence, the beneficial effects of catechins may be achieved through a wide range of molecular mechanisms as reviewed in the following sections (Table 1).

#### 3.2.1. Huntington Disease (HD)

HD is caused by an unstable polyglutamine (polyQ) repeat enlargement (which must exceed a critical limit of ~35 repeat units) within the first exon of the IT-15 gene that encodes the 350 kDa huntingtin (htt) protein [64]. The extent of htt fibril aggregates prompts gradual damage of cortical and striatal neurons and formation of neuronal inclusions containing aggregated htt protein [65]. A predominant symptom of HD is chorea, characterized by fidgety movements. Tetrabenazine (TBZ) has shown to be effective in the treatment of chorea; it acts by blocking the vesicular monoamine transporter 2 (VMAT-2) in the CNS, reducing dopamine levels. Antipsychotic drugs have also been used [66]. Cognitive improvement has been shown with cholinesterase inhibitors (rivastigmine) for the treatment of progressive HD-related dementia, although further placebo-controlled trials are needed [67]. Over the past years, some studies have focused their attention on molecules that may inhibit the aggregation of mutant htt. EGCG and other compounds from green tea have been identified as strong suppressors of htt exon 1 aggregation. Ehrnhoefer et al. used in vitro models of HD and demonstrated that green tea polyphenols may regulate early stages of polyQ expansion, therefore blocking the formation of amyloid fibrils processes. The beneficial properties of EGCG and its products, e.g., removal of free radicals, reduction of reactive oxidative species, or chelation of bonding metal ions, may help decrease htt accumulation and reduce the toxic effects in in vivo HD models [52]. The authors conclude that EGCG suppresses polyQ aggregation and may preserve neuronal cells expressing an altered htt protein from its detrimental effects. Moreover, lipid composition alterations in cellular and subcellular membranes prompts the accumulation of amyloid-producing proteins. Furthermore, modified htt showed improved affinity to phospholipids if compared to non-mutated htt, leading to alteration of phospholipid double layer stability. In a study by Beasley et al., the authors analyzed if the presence of lipid vesicles changed the ability of EGCG or curcumin to modify aggregated htt and affect htt and lipid linkage. They observed that, regardless of the interaction with membrane environment, EGCG prevented htt fibril formation if lipid vesicles were present, inhibiting amyloid formation [51]; this shows that EGCG is a potential drug candidate for HD treatment and of other conditions involving altered protein aggregation linked to amyloid deposition. A recent study in Drosophila models show that EGCG improves neurodegeneration caused by mutant htt [53]. An ongoing clinical trial in Germany is evaluating the changes in cognitive function in HD patients by administering a maximal daily dose of 1200 mg of EGCG compared to placebo for 12 months [68]. Although preliminary results are promising, more clinical trials in humans are needed to assess the beneficial effects of EGCG in HD.

#### 3.2.2. Multiple Sclerosis (MS)

MS is a chronic autoimmune disease that affects the CNS. It characterizes by focal lymphocytic (T-cells) infiltration, leading to inflammation and demyelination, and proliferation of astroglia with neuronal damage. It can cause many different neurologic symptoms, such as sensory issues, movement problems, and visual impairment. There are different clinical forms of MS, the most common being relapsing-remitting MS (RRMS), distinguished by relapses with new symptoms and a posterior partial or total recovery. Some people affected by RRMS develop secondary progressive MS (SPMS) over the years, characterized by continued disability progression, with or without remission periods. Primary progressive MS (PPMS) is characterized by a progressive course from onset [69]. Currently, there is no curative treatment for MS. However, there are many disease-modifying therapies (DMT) that may help reduce the relapses and magnetic resonance (MRI) activity of the disease [70].

Different animal models allow studying molecular and clinical aspects related to MS, being experimental autoimmune encephalomyelitis (EAE) one of the most well established due to the similarities with human MS clinical, immunological, and neuropathological features. Using this animal model, EGCG seems to attenuate EAE and reduce the severity of its symptoms, diminishing immune cell infiltration, modulating T-cell balance [59,60], and reducing inflammatory cytokines (IL-6, TNF-α, interferon-gamma, and IL-17) [59,60]. Other authors evaluated a combined treatment using glatiramer acetate (GA), a DMT, and EGCG in EAE. Their results showed significant delay of disease onset, reduced clinical severity, and reduced inflammatory infiltrates [61]. Other studies used a cuprizone-induced MS mice model to test the effects of EGCG. EGCG significantly increased the proteolipid protein and oligodendrocyte transcription factor 1 in the cerebral cortex, which is related to the remyelination process [62,63]. Both animal models showed that EGCG treatment improves clinical symptoms or molecular mechanisms of the affected mice.

Few studies have assessed the effect of catechins on human populations, most of which are on RRMS. Bellman–Strobl et al. evaluated the efficacy and safety of EGCG + GA on 122 RRMS patients (less than 80% finished the study). Their efficacy endpoint was the development of new hyperintense lesions on T2-weighted brain MRIs. The 18-month treatment with 800 mg/day oral EGCG added to GA showed no superiority in MRI findings and clinical activity, compared to placebo [54]. A double-blind clinical trial analyzed the mechanisms of antioxidant therapy on MS patients. Results revealed that patients with RRMS treated with GA + 600 mg of EGCG had lower nitrogen oxide (NOX) levels in CD11b+ monocytes than patients treated with GA or untreated patients, which shows improvement on oxidative stress level with EGCG treatment [56]. Mähler et al. found that a daily intake of 600 mg of EGCG for 12 weeks improves muscle metabolism during exercise on RRMS patients [57]. Another group concluded that after 36 months of daily administration of EGCG or placebo, there were no significant inter-group differences in brain atrophy on PPMS or SPMS assessed with MRI parenchymal brain function (PBF) evaluation. Researchers wondered if the negative outcome of the study may be a consequence of the small sample size and a dropout rate of more than 30% [55]. Controversially, Lovera et al., evaluated the efficacy of Polyphenon E (green tea extract with 50% of EGCG) in MS patients. They found that the administration of 800 mg of EGCG on a daily basis for six months increased N-acetyl aspartate levels in the brain, revealing a neuroprotective effect; however, the study had to be stopped because five out of seven patients on the Polyphenon E group had abnormal liver function tests and the researchers concluded that Polyphenon E may increase the risk of hepatotoxicity [58]. In other reviewed articles, safety of EGCG treatment was assessed, concluding that it was a safe medication with similar adverse events in the placebo and treatment groups [54,55]. Polyphenon E contains other polyphenols besides EGCG and may have small amounts of caffeine, metabolized in the liver, which may explain these safety differences. Therefore, efficacy of catechins in MS patients is controversial and not all the reviewed articles show improvement with catechin treatment. Possible explanations include small sample sizes, heterogeneity in the studied groups, differences in EGCG administered doses, and different evaluated outcomes.

### 3.3. Catechins in Fetal Alcohol Spectrum Disorders (FASD)

Prenatal exposure to ethanol causes a set of disabilities known as FASD. The most serious form is fetal alcohol syndrome (FAS), characterized by facial dysmorphology, neurobehavioral impairment, and growth deficiency [71]. Alcohol consumption has several pathological effects, such as dysregulation of the neuroimmune system, neurotransmitter disorders, or epigenetic modifications [72]; however, oxidative stress damage is considered one of the primary causes of alcohol keratogenesis [73]. Although early therapies, such as developmental therapy or behavioral interventions, may prevent some specific disorders and secondary disabilities [74,75], there are no specific treatments for alcohol-related alterations, and FASD phenotypes persist for a lifetime. Pharmacotherapy for the treatment of specific cognitive and behavioral traits that share a common framework with other neurological disorders has been tested. Exercise improves brain function and nutritional interventions focus on nutritional deficits (vitamins and minerals), oxidative stress (vitamin C, vitamin E, omega-3 fatty acids), and epigenetic modifications (choline, betaine, folic acid, methionine, zinc) produced by maternal alcoholism [76]. The ability of catechins to penetrate different organs and their antioxidant properties directs the attention to these molecules as candidates for FASD treatment [77]. Four preclinical studies on potential EGCG therapy were found in the search (summarized in Table 2), although none involved humans.

EGCG reduces oxidative-nitrosative stress and enhances antioxidant defense [79,80,81]. Research in murine models showed increases of glutathione and superoxide dismutase levels and mitigation of lipid peroxide and nitrite levels with EGCG treatment at 50 or 100 mg/kg in rat neonates [80], as well as reduction of hydrogen peroxide (H_2_O_2_) and malondialdehyde (MDA) production in pregnant mice treated with 400 mg/kg EGCG on gestational days (GD) 7–8 [79]. Conversely, prenatal alcohol exposure (PAE) and EGCG treatment at 30 mg/kg showed EGCG-related reduction of Nfr2 [81], probably because EGCG mainly exerts its antioxidant action through other molecular pathways such as induction of phase II detoxifying enzymes and scavenging of ROS.

Regarding fetal growth, EGCG regulates placental angiogenesis disorders and fetal growth restriction produced by PAE by maintaining adequate placental vascularization [79,81], as evidenced by the compensatory effect on the expression of vascular endothelial growth factor-A (VEGF-A) and vascular endothelial growth factor receptor 1 (VEGF-R1) in a mouse model of continuous ethanol exposure [81].

Additionally, EGCG can cross the blood-brain barrier, penetrating the brain [77]; thus, treatment with this molecule has shown favorable results on fetal brain development processes affected by ethanol. EGCG inhibits neuronal apoptosis produced by ethanol, as showed by decreased apoptosis in an in vitro model with rhombencephalic neurons in culture from rat fetuses [78] and reduction of caspase 3 and NF-κB in a rat model [80]. Tiwari et al. report a decrease in acetylcholinesterase activity, maintaining adequate levels of acetylcholine, an inductor of glial cell proliferation that acts as a trophic factor by preventing neuronal apoptosis [80]. Reduction in apoptosis may prevent microcephaly and PAE-related cognitive disorders. Apart from the anti-oxidant and anti-apoptotic effects, EGCG can ameliorate ethanol-mediated inflammatory responses, as showed by decreases of TNF-α and IL-1β levels [80]. Moreover, outcomes from a study in a mice model of continued PAE determined that a daily dose of 30 mg/kg of EGCG could help prevent losses of mature neurons and maturation delay produced by ethanol, as indicated by changes in neuronal nuclei (NeuN) and doublecortin (DCX) expression, respectively. Similarly, EGCG up-regulates glial fibrillary acidic protein (GFAP) and brain-derived neurotrophic factor (BDNF) by compensating for early astrocyte differentiation and disturbances in neuronal plasticity generated by maternal drinking [81].

Recent evidence from an analysis of genome-wide DNA methylation patterns in a cohort of 206 children suggests that PAE is associated with different DNA methylation patterns [82], including reduction in DNA methyltransferase 1 (Dnmt1) expression [83]. Nevertheless, an in vitro model demonstrated that tea catechins (catechin, EC, and EGCG) inhibited DNMT1-mediated DNA methylation, with EGCG being the most potent suppressor. Additionally, the same effect was observed in cultured cancer cells [84]. In our bibliographic search, no studies assessing the effect of EGCG on DNA methylation pattern in FASD were found; it would be interesting to analyze if the effect of catechins on methylation patterns in fetal cells differs from that in somatic cells.

**Figure 3:**
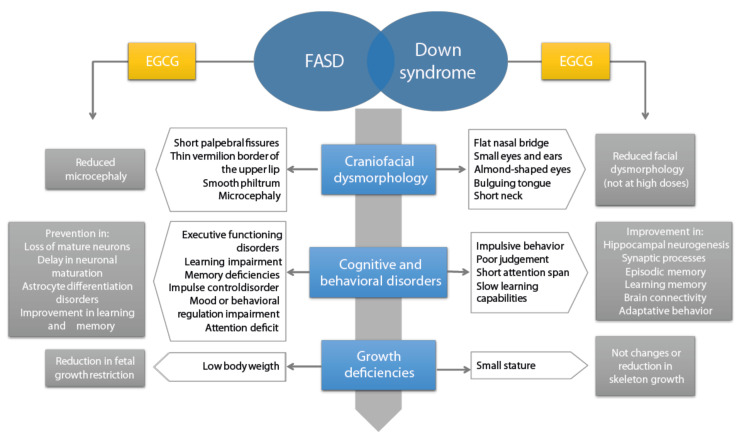
Common pathophysiological characteristics of Fetal Alcohol Spectrum Disorders (FASD) and Down syndrome, and effect of epigallocatechin-3-gallate (EGCG) on the different phenotypes.

Finally, the aforementioned disturbances in fetal neurodevelopment lead to behavioral and cognitive disorders, such as memory and learning impairments, attenuated by EGCG treatment [80]. EGCG modulates the expression of genes and proteins involved in brain development and neural differentiation, like Otx1 (orthodenticle homolog 1 [Drosophila]) and Sox2 (sex determining region of the Y chromosome [Sry]-related high mobility group box2) [79]. Interestingly, FASD and DS share common characteristics, as shown in Figure 3. EGCG inhibition of the overexpressed dual-specificity tyrosine-(Y)-phosphorylation-regulated kinase 1A (Dyrk1A) protein has shown benefits in neuronal plasticity processes in DS patients [85], which may be another potential molecular pathway to study in FASD and a therapeutic target for cognitive disorders.

Results from FASD-like rodent models have to be tested in humans. The neuro-SAF clinical trial was designed to assess the efficacy of EGCG (9 mg/kg/day) on cognitive performance in a cohort of 40 FAS children aged 7 to 14 years [86]. Preliminary outcomes, after six months of treatment, show improvements in working memory and recent and delayed memory. Future outcomes from this and other clinical trials are necessary to translate the encouraging results obtained in animals to humans.

### 3.4. Effects of Catechins on Down Syndrome (DS) Neurocognitive Disorders

DS is the most common genetic condition related to intellectual impairment. It is caused by the presence of a third copy of chromosome 21 and characterized by typical facial features such as a flattened face, epicanthus, up-slanted eyes, and hypotonia [87]. Cognitive deficits in DS may express different degrees of severity, deriving from the imbalance between increased suppression of synapsis in the hippocampus and strong stimulation in the cerebral cortex [87]. Recent studies have focused on synaptic dysfunction including changes in synaptic proteins, such as modifications in receptors for glutamate, GABA, or other neuromodulators, and centered on reduced synaptic function at various locations and structures [88]. DS may lead to failure in adaptation to surrounding requests, with impaired plasticity in the response to the environment. The main outcomes are transformations of the substantial composition of synapses, which impairs the information process and storage capacity of neural networks. Neuropsychological features are characterized by evident hippocampal-dependent disabilities affecting in particular spatial memory, and decreased levels of cognitive skills and abilities [89]. Treatments for minimizing dementia in patients with DS have been proposed. Psychotropic drugs, antipsychotics, and antidepressants have been used in DS with poor results [90]. Acetylcholinesterase inhibitors have been studied in DS dementia for their neuroprotective properties, but there is lack of evidence to support their efficacy and further trials should be conducted [91,92].

During the last years, EGCG therapy has been proposed for DS due to its pro-cognitive effects [93]. Table 3 summarizes the reviewed studies. Recent research suggests that EGCG exerts its beneficial effects through its ability to suppress metalloproteinase 9 (MMP-9). In DS, the metabolic route of nerve growth factor (NGF) is impaired, deregulating enzymes involved in proteolysis such as MMP-9. Therefore, EGCG could modulate the degradation function of MMP-9 on NGF [94]. In addition, the most recent studied mechanism of EGCG focuses on the inhibition of DYRK1A, a serine/threonine kinase involved in cell growth and neuronal enhancement and plasticity; DYRK1A is overexpressed in DS and considered the main cause of cognitive dysfunctions in these individuals. Studies in transgenic mice overexpressing DYRK1A have demonstrated that low, intermediate or high dose of EGCG is well distributed in the brain without adverse effects, and the favorable mechanisms derived from its actions are related to the interconnection and inhibition on DYRK1A [50,95]. Therefore, the intervention on the concentration of active DYRK1A is a target for DS therapy and may rescue behavioral difficulties acting on GABAergic and glutamatergic routes [95].

Some studies in Ts65Dn mouse models have shown that environmental enrichment (EE) associated with EGCG treatment at an average dose of 30 mg/kg/day enhances learning and memory in the cortex and hippocampus, preventing cognitive degeneration [96,100] and at 42 mg/kg/day rescues phosphoprotein deregulation in the hippocampus, restoring the epigenetic profile and reversing the kinome deregulation process. These mechanisms may promote the cognitive improvement induced by green tea derivatives [105,106]. Other experimental studies analyzing cell cultures or neural progenitor cells (NPCs) isolated from the hippocampus of Ts65Dn mice, revealed that EGCG treatment reactivated mitochondria bioenergetics and biogenesis, severely compromised in DS, and promoted neuronal progenitor cell proliferation with helpful effects on neuronal plasticity impeding the production of ROS [101,102]. EGCG treatment during the neonatal period may be more efficient in modulating disabilities in DS. However, it should be considered that EGCG does not cause long-term effects on hippocampal physiology [103].

Another study with an animal model demonstrated impaired growth in both euploid and trisomic mice with daily EGCG gavage treatments (200 mg/kg/day) over three weeks; EGCG did not show positive effects on conduct assessment in Ts65Dn mice [97]. Similarly, neither lower EGCG (20 mg/kg/day for three weeks) doses, nor higher doses for longer periods (50 mg/kg/day for seven weeks), initiated during adolescence, improved cognitive impairment in DS mouse models [98,99]. A plausible explanation for the different results may be the distinct environmental condition of the experiments, the different types of mouse models, or the non-uniformity in the design of cognitive stimulation. However, the controversy of such results highlights the importance in determining the correct EGCG dosages to consistently improve DS phenotypes, connecting those properties to the effects of EGCG (or supplements included in EGCG) in precise markers at cerebral and skeletal levels. Furthermore, the developmental timing at beginning treatment may play a critical role, combined with the effects of other substances comprised in some EGCG compounds.

To the best of our knowledge, published double blind, placebo-controlled clinical trials in humans with DS are scarce. Two studies were performed by the same research group (de la Torre and collaborators) in a DS population aged 16–34 years. The authors demonstrated that EGCG at 9 mg/kg/day given alone during six months or combined with cognitive training for 12 months, significantly improved visual remembrance and memory, response inhibition, and conduct performance in comparison with placebo alone or associated with cognitive training [85,104]. The main effect of EGCG was the enhancement of immediate detection memory. Thus, we can speculate that by normalizing DYRK1A activity, EGCG may be helpful if associated with cognitive training, which also improves plasticity. In humans, plasma homocysteine, a biomarker of hippocampal DYRK1A levels is related to memory improvement, suggesting close association of intellectual progress with DYRK1A activity [104]. Another recent trial showed that EGCG modulates facial development with dose-dependent effect if administered at low doses (30 mg/kg/day) before the age of three. Higher doses (100 mg/kg/day) would potentially have detrimental effects [107].

Therefore, treatment with EGCG is a new approach for DS management that targets synaptic- and plasticity-related mechanisms implicated in learning and memory to help improve cognitive disabilities associated to this condition. Further research on the effectiveness and safety of EGCG are needed, as well as reviews on the management in combination with other nutritional supplements.

### 3.5. Neurologic Effects of Catechins on Healthy Populations

Over the last years, some studies have investigated if catechins can improve cognitive function in healthy populations (Table 4). Two authors assessed the association between the level of dietary intake of catechins and other flavanols. Kesse–Guyot et al., registered intake questionnaires and evaluated cognitive function 13 years later on 2574 adults. They found an association between catechin intake and improvement of language and verbal memory score and lower scores on executive functioning factor [108]. On the same line, Biasibetti et al., observed an inverse association between dietary intake of catechins and impaired cognitive status in a population of 808 Italian individuals aged over 50 years [109]. Other researchers evaluated the effect of EGCG on mood and brain activity in healthy adults. Scholey et al., showed a significant increase in alpha, beta, and theta electroencephalogram activity in midline frontal and central regions, as well as an increase of self-rated calmness and a reduction of stress after the administration of 300 mg of EGCG 94% 120 min before the test [110]. On the other hand, Emma et al., evaluated if 135 mg and 270 mg of EGCG affects cognitive performance, mood, and cerebral blood flow (CBF) in humans; their results showed no significant differences on cognitive performance and mood, but there was a reduction of CBF in the frontal cortex when 135 mg of EGCG were given [111]. Other authors evaluated the effect on the brain function of different products with a high catechin content. Liu Y et al., examined the effect of 5.4 g of decaffeinated green tea extract on working memory in 20 healthy young and old women on a single blind, placebo-controlled crossover study, performing working memory tests after the intervention. Results showed an improvement in reading span only in the older group; no other differences were observed [112]. Dietz et al., investigated the effect of matcha tea on cognitive performance and mood in a randomized, placebo-controlled, single blind, crossover study using 4.0 g of matcha tea (containing 280 mg of EGCG) and placebo. Results suggest that matcha tea may induce slight effects on episodic secondary memory and speed attention [113]. Moreover, Mohamed et al., evaluated the cognitive effects of oil palm leaves (OPLE), rich in antioxidant catechins, in adult humans, as well as in a rat model. Results in human volunteers consuming 500 mg of OPLE/day for two months showed significant improvement on short-term memory, processing speed, and spatial visualization. In the rat model, OPLE revealed neuroprotection on nitric oxide (NO) deficient rats [114]. All these findings suggest that catechin use in healthy populations aged between 20 and 60 years promotes improvement of cognitive function. Further investigation is needed to confirm these results and establish the molecular and cognitive pathways of these potential benefits.

#### Age-Related Cognitive Decline and Catechins

Age-related cognitive decline refers to normal mental decrease with increasing age in people who do not meet the criteria for dementia or mild cognitive impairment (MCI). Risk factors associated to its development are genetics [119,120], certain medical conditions, diet, and lifestyle [121]. There are no specific treatments for age-related cognitive decline and the use of catechins is increasingly gaining interest for its prevention and treatment. In the reviewed studies different green tea products were used in blind, randomized controlled studies. Ide et al. evaluated the effect of 2.0 g/day of green tea (220.2 mg of catechins) given for a period of one year to older patients with cognitive dysfunction. No significant inter-group changes in the Mini-Mental State Examination (MMSE) scores were observed, but serum level of malondialdehyde-modified low-density lipoprotein, an oxidative stress marker, was lower in the green tea group [5]. Moreover, Sakurai et al. assessed the effect of three grams of powder from fresh matcha for 12 weeks administered to an elderly population without dementia or with MCI; no significant differences were seen in cognitive, memory and impulsivity tests. However, when only women were analyzed, differences were obtained between the matcha and placebo sub-groups with the Montreal cognitive assessment (MoCA) tool [116]. Similarly, a study by Baba et al. included volunteers between 50–69 years with self-assessed cognitive decline (MMSE > 24 score) who were given green tea catechin capsules (336.4 mg) or placebo on a daily basis for 12 weeks; significant improvement on working memory tasks were observed in the group who received green tea, evaluated with the Cognitrax battery of tests. No other differences on the tests or blood biomarkers were revealed [115]. Another study aims to evaluate the efficacy of an intervention in lifestyle combined with EGCG on the prevention of cognitive decline; no results have been published yet [122]. Some studies with animal models evaluate the effect of catechins on cognitive decline. Uno et al. used senescence-accelerated prone 10 (SAMP10) mice to examine how much green tea catechin is needed to prevent age-related cognitive decline. Their findings revealed significant improvement of long-term memory with 60 mg/kg catechin intake and improvement of working memory with 30 mg/kg. [117]. Similarly, Ramis et al. used old male rats to evaluate the effect of green tea extract on brain and cognitive status on elderly populations. After 28 days of treatment the rats showed significant improvement on working memory and episodic memory [118]. Different molecular mechanisms are involved, e.g., decrease in hippocampal neuroinflammation by modulating the protein sirtuin 1 (SIRT1) [118] or the increase expression of the Nr4a, Fos, Egr1, Npas4, Cyr61 genes, involved in long-term changes of neuronal circuits and plasticity synapses [117]. These works reveal that although catechin administration in animal models seem to have promising effects on age related cognitive decline, its prevention or improvement remains uncertain on the human population. Further evidence in needed to determine which areas of cognition can be improved with catechin supplementation and the populations that will benefit from this intervention.

## 4. Discussion

Interest in catechin use has been growing in recent years. Their natural origin and multiple mechanisms of action make them a feasible option for the management of different neurological diseases. Although flavan-3-ols or catechins comprise a high number of bioactive compounds, this study focused mainly on EGCG. It has potent antioxidant activity compared to other catechins, positive effects on brain function, and ability to cross the blood-brain barrier, making it a promising tool for the treatment of neurodegenerative disorders. Modulation of microglia activation, reduction of inflammatory mediators [31,32,34], iron-chelating properties [8], neuritogenic activity [37], autophagic flow restoration, and reduced apoptosis [45,48], are some examples of how EGCG can act on brain function. The use of EGCG in less common neurodegenerative diseases, such as HD, is supported by the ability of EGCG to inhibit protein aggregation [51,52,53]. Preliminary results from clinical trials assessing changes in cognitive performance are promising [68], but further clinical trials are required in humans to validate them. Neuroinflammation is characterized by microglial activation, which contributes to HD progression; thus, this flavan-3-ol may be used to reinforce regular treatments to help decrease inflammatory and apoptotic mediators in microglia and exert a neuroprotective effect. The immunomodulatory effects of EGCG also play a critical role in MS, characterized by focal lymphocytic (T-cells) infiltration, demyelination, and axonal and neuronal damage. EGCG diminishes immune cell infiltration, modulates T-cell balance [59,60], and reduces inflammatory cytokines [60], attenuating the symptoms of the disease. Furthermore, EGCG interferes in the modulation of neuronal transcription factors -important in remyelination processes [62,63]- and reduces oxidative stress [56]. Although catechins show no effect on the development of new hyperintense lesions on MRI [54] or brain atrophy, their use has demonstrated improvement of muscle metabolism during exercise [57] and amelioration of neurodegeneration, as judged by the increase of brain N-acetyl aspartate levels in MS individuals [58].

Catechins, particularly EGCG and ECG, exert their neuroprotective effects through an antioxidant action via free radical scavenging and regulation of oxidative stress response [26,30]. EGCG has a greater ability to donate electrons in comparison to other flavan-3-ols due to its eight hydroxyl groups, notably in the 3′, 4′, and 5′ positions, and its antioxidant potential mainly comes from these functional groups. Moreover, its phenolic groups (particularly in the B-ring) can chelate metals, increasing its antioxidant capacity. EGCG can also reduce certain metals, such as iron and copper, related to the Fenton reaction, an advanced oxidation process in which highly reactive hydroxyl radicals (OH-) are produced [123].

EGCG´s antioxidant properties are key for early interventions aiming to prevent secondary FASD disabilities, since one of the main pathophysiological mechanisms of alcohol-related disorders is oxidative stress. EGCG modulates antioxidant defense and oxidative stress balance in FASD-like rodent models [79,80,81], resulting in the reduction of neuronal loss and recovery of maturation delay, and prevents early astrocyte differentiation and disturbances in neuronal plasticity produced by maternal drinking [81]. Moreover, EGCG prevents neuronal apoptosis and ameliorates inflammatory response secondary to PAE [80], and regulates the expression of genes and proteins involved in brain development and neural differentiation [79] associated with improvements in memory and learning abilities [80]. Understanding the effect EGCG has on ethanol-induced epigenetic alterations, as well as the modulatory effect of EGCG on DYRK1A may clarify the beneficial outcomes of catechins on FASD neurodevelopment. Regarding fetal growth, EGCG promotes the correct development by regulating placental angiogenic processes [79,81]. Studies in children are necessary, such as neuro-SAF, to evaluate the effect of EGCG on cognitive performance and translate the promising results found in animals to human populations [86].

As FASD and DS show common molecular and cellular origins during fetal development, EGCG treatment has been proposed to reverse disabilities related to both syndromes by improving neurogenesis, neuronal differentiation, cell death, and synaptic plasticity processes by regulating Dyrk1A overexpression [49]. Recent literature demonstrates a rescue of behavioral and cognitive outcomes in DS animal models treated with EGCG supplements. EGCG inhibits DYRK1A overexpression, a gene involved in a range of routes associated to neural progenitor cell growth, and the primary gene candidate to explain DS phenotype [124]; treatment with EGCG may normalize its overproduction improving behavioral and neural phenotypes in this condition. EGCG acts on hippocampal neurogenesis, responsible of cognitive disabilities [88]; thus, treatments aiming to restore neurogenesis alterations may be effective in DS individuals [102]. Moreover, recent studies highlight that EGCG modulates epigenetic changes, restoring epigenetic balance [106] and promotes mitochondrial biogenesis [101]. The potential effect of EGCG in modulating plasticity alterations in DS seems to be more beneficious if the treatment if started during the first years of life [106].

Furthermore, the combination of cognitive training with EGCG may synergistically improve the effects of such therapy in FASD and DS [96].

Finally, the antioxidant properties of catechins and other flavanols on healthy populations may help prevent many chronic diseases related with lifestyle as judged by significant increase in alpha, beta and theta electroencephalogram activity in the midline frontal and central regions [110]. Observational studies have shown that regular diets rich in catechins improve different cognitive functions [108,109]. Thus, some authors have developed randomized, placebo-controlled studies to assess the use of EGCG or green tea extracts. Their findings suggest that catechins may promote improvement in cognitive function of healthy populations [110,111], but further investigation is needed to confirm the results and establish the molecular and cognitive pathways of these potential benefits. The reduction in hippocampal neuroinflammation or the increased expression of genes involved in long-term changes in neuronal circuits and plasticity synapses may be the mechanisms associated to the beneficial effects exerted by EGCG on age-related cognitive decline [117,118]. To date, there are no studies in humans reproducing the encouraging results obtained with the use of EGCG in animal models. Human research has been carried out with different types of green tea extracts at different proportions of many catechins, and mild improvements have been seen in certain areas of cognition or population subgroups [5,115,116]. Thus, prevention or improvement of cognitive decline remains uncertain, requiring more evidence to determine which populations and areas can be improved with catechin supplementation.

## 5. Conclusions

A large amount of evidence suggests that catechin intake is beneficial for managing neurological and neurodegenerative disorders. EGCG, the most commonly used catechin, has antioxidant, anti-inflammatory, neuritogenic, and anti-apoptotic properties. Common molecular mechanisms in certain neurologic pathologies make EGCG a feasible therapeutic option, administered alone or in combination with other interventions. However, mores studies, particularly in humans, are needed to further extend the knowledge on catechin involvement in the different molecular pathways related to neurological disorders and their clinical manifestations. Clarifying the contribution of a wide range of polyphenols contained in green tea extracts and duration of catechin beneficial effect is necessary. Future research in this field should focus on the modulation of neuronal mechanisms linked to degenerative disorders and target brain regions involved in neuronal failure. Cognitive training combined with pharmacological therapies would open a range of great opportunities in order to rescue the degeneration of neuronal activity typical of such diseases. Studies in humans must focus on the assessment of the average dose related to beneficial outcomes without adverse effects. Further studies must elucidate the epigenetic restoration properties of EGCG and its modulator effects on the production of ROS, which increase during ageing and are responsible of oxidative stress, causing the alterations in the epigenetic balance of the cell [125].

## Figures and Tables

**Table 1:** Catechin use in neurodegenerative disorders.

Author (Year)	Objective	Type of Study and Sample Size	Inclusion and Exclusion Criteria	Interventions	Methodology	Main Outcomes	Conclusions	Quality of Evidence
**Huntington disease**								
**Beasley et al., 2019** [51]	To investigate the ability of EGCG to inhibit aggregation in the presence of lipid vesicles (POPC or TBLE)	In-vitro model	Glutathione S-transferase (GST)-htt-exon1(46Q) fusion protein purified from *E. coli*	EGCG co-incubation with -exon1(46Q)	POPC or TBLE lipid vesicles preparation. ThT aggregation assays Image acquisition by atomic force microscopy Vesicle-binding assay	↓ exon 1(46Q) aggregation (*p* < 0.01) ↓ exon 1(46Q) fibril formation and aggregation in the presence of lipid vesicles (POPC or TBLE) (*p* < 0.01)	EGCG inhibitory effect on htt aggregation process persists in the presence of lipid vesicles	++
**Ehrnhoefer et al., 2006** [52]	To analyze the dose-dependent effect of EGCG on mutant htt exon 1 protein aggregation	In-vitro model	Yeast cultures and transgenic HD flies overexpressing a pathogenic htt exon 1 protein	Mutant GST-tagged htt exon 1 fusion protein with 51 glutamines (GST-HDQ51) was incubated with green tea polyphenols (GCG, GC, EGC, and EGCG)	Dot blot assays Atomic force microscopy studies	↓ mutant htt exon 1 protein aggregation, polyQ-mediated htt protein aggregation and cytotoxicity ↓ photoreceptor degeneration ↑ motor function	EGCG acts as modulator of htt exon 1 misfolding and oligomerization reducing polyQ-mediated toxicity in vivo	++
**Varga et al., 2018** [53]	To study the effects of green tea on HD pathogenesis	Experimental transgenic *Drosophila* model of HD *n* = 356 (HttQ93on CM), *n* = 345 (HttQ93 on GTM), *n* = 122 (HttQ20on CM), *n* = 118 (HttQ20 on GTM)	Inclusion: Neuron-specific GAL4 driver strain *w P{GawB}elavC155* males and *w; UAS-Httex1p-Q93* or control *w; UAS-Httex1p-Q20* females	*Drosophila* strain exposure to green tea medium	Eclosion, survival, climbing assay and pseudopupil tests Polyphenol content determination by Folin-Ciocalteau method Huntingtin protein level measurement by Immunoblot	Mutant huntingtin expressing Drosophila exposed to green tea presented: =Viability (*p* < 0.001) ↓ Neurodegeneration (*p* < 0.001) ↑ Longevity (*p* < 0.001)	Green tea consumption might modulate symptoms of HD.	++
**Multiple sclerosis**								
**Bellmann-Strobl et al., 2021** [54]	To evaluate the safety and efficacy of EGCG + GA in RRMS patients	Prospective, double blind, Ph II, randomized controlled trial. *n* = 122	Inclusion: age: 18–60, EDSS score 0–6.5, stable with GA. Exclusion: other forms of MS, major diseases, laboratory abnormalities, other medication	800 mg oral EGCG/day or placebo for 18 months	Neurologic assessments, safety monitoring, laboratory exams and MRI at baseline and every three months	No differences with EGCG + GA treatment on brain MRI and on SAE and AE in comparison to placebo + GA.	No superiority of EGCG + GA compared to placebo + GA in MRI changes or clinical disease activity. EGCG at 800 mg/day was safe	++++
**Rust et al., 2021** [55]	To evaluate if treatment with EGCG affects progression of brain atrophy and its safety on primary and SPMS	Prospective, double blind, phase II, randomized controlled trial. *n* = 61	Inclusion: age: 18–65, EDSS score: 3–8, relapse-free period of minimum 30 days. No MS modifying therapy. Exclusion: RRMS, major diseases, laboratory abnormalities, hepatotoxic medications.	Increasing doses of EGCG until reaching 1200 mg or placebo, for 36 months	Neurologic assessments at baseline and every six months. Safety monitoring and laboratory exams every 2–3 months. MRI at baseline and every year evaluating PBF	No differences on PBF decrease, SAE and AE between groups.	No differences on EGCG group didn’t on brain atrophy compared with placebo. EGCG 1200 mg/day was safe	++++
**Mossakowski et al., 2015** [56]	To investigate the role of oxidative stress on neuronal degeneration and on antioxidant therapy	Murine and human: *n* = 6. Groups: RRMS GA, RRMS GA + EGCG, RRMS untreated, CIS, controls, SPMS)	Patients with RRMS, SPMS, or CIS	EGCG 600 mg or placebo in groups of RRMS + GA. EAE murine model of MS	Examine the oxidation of NADH and NADPH in mononuclear cells with a two-photon laser-scanning microscopy to see activation of NOX enzymes	EGCG + GA ↓ NOX in CD11b + monocytes in MS and EAE.	EGCG counteracted NOX overactivation in MS patients	++
**Mähler et al., 2015** [57]	To investigate if EGCG improves energy metabolism and substrate utilization in MS	Randomized, double blind, placebo-controlled, crossover trial *n* = 18 (eight men)	Inclusion: RRMS, treated with GA for six months. EDSS score: 4.5. Age: 20–60. BMI: 18.5–30.0. Exclusion: other MS forms. Relapses three months before or during the study. Other diseases. Regular caffeine or green tea intake, social drugs	EGCG (600 mg/day) and placebo 12 weeks (four weeks of washout).	Measurements from blood samples, microdialysates from adipose tissue and skeletal muscle, fasting and postprandial EE, FAOx and CHOx rates, at rest or during 40-min of exercise	Working efficiency: placebo: 20 +/− 3 EGCG: 25 +/− 6 (*p* = 0.004) Postprandial FAOx: Placebo: 8.3 +/− 4.3 EGCG: 8.6 +/− 5.0 (sex differences)	EGCG given to MS patients over 12 weeks improves muscle metabolism during moderate exercise, predominantly in men	++
**Lovera et al., 2015** [58]	To evaluate the safety and futility of Polyphenon E treatment	PhI: single group. PhII: randomized double blind placebo-controlled study. *n* = 10 on phI *n* = 13 on phII	Inclusion: MS with RRMS or SPMS. six-month stability. Treatment with GA or no treatment in PhI, GA or Interferon β in PhII. Exclusion: Bone marrow ablation or alemtuzumab use. Mitoxantrone, cyclophosphamide, natalizumab or fingolimod use during the past nine months. Other diseases	PhI: Polyphenon E capsules (400 mg of EGCG) twice daily for six months. PhII: Polyphenon E or placebo, same dose for one year	Measure plasma levels. Evaluate adverse events monthly. Evaluate NAA levels at 0, 6, and 12 months using MRI	Polyphenon E: ↑ NAA adjusted creatinine. 5/7 participants had elevated liver enzymes in Ph 2	400 mg of EGCG/12 h olyphenon E increased NAA brain levels. Polyphenon E may increase the risk of hepatotoxicity	+++
**Wang et al., 2012** [59]	Determine the effect and mechanisms of EGCG on EAE development.	Experimental animal model. Four groups, *n* = 12/group	Specific pathogen-free C57BL/6 female mice.	Diet supplement with 0%, 0.15%, 0.3%, or 0.6% EGCG 30 days, after produce EAE.	Signs were daily scored from day 0 to 30 after EAE induction. Euthanasia and histology and molecular evaluation.	EGCG ↓ symptoms and pathological features in the central nervous system.	EGCG may attenuate EAE autoimmune response.	+
**Sun et al., 2013** [60]	Investigate the mechanism of EGCG on amelioration of EAE	Experimental animal model *n* = 10/group.	Male C57BL/6 mice, 7 weeks old	EAE induction. When clinical signs start: 300 μg EGCG in 100 μL PBS daily or PBS alone.	Clinical signs evaluation. After death histopathology and molecular evaluation.	EGCG ↓ disease severity in EAE, ↓ brain inflammation and ↓ demyelination damage.	EGCG may be useful for the MS treatment.	+
**Herges et al., 2011** [61]	Evaluate the effect of GA and EGCG in vitro and in a EAE model.	Experimental animal model and in vitro. *n* = 8/group	6–8 week old female SJL/L mice	Prevention and treatment with EGCG 300 μg/12 h or vehicle and 50–150 μg GA/24 h from day 9 before EAE production.	Valuation of clinical signs and histological examination.	EGCG + GA ↓ disease onset, ↓ clinical severity and ↓ inflammatory infiltrates.	GA + EGCG may be useful and safe approach for MS.	+
**Semnani et al., 2016** [62]; Semnani et al., 2017 [63]	Study EGCG effects on the PLP and Olig1 expression.	Experimental animal model. *n* = 60 (6 groups, *n* = 10)	C57BL/6 male mice, 8 weeks old.	Induction demyielinization with cuprizone. Injection of EGCG 50 mg/Kg/day, PBS, or nothing.	After 2 or 4 weeks, euthanasia and cerebral exam Western Blot or Real-time PCR	EGCG: ↑ PLP and Olig1 expression	EGCG increases PLP and Olig1 expression in the cerebral cortex of this mouse model of MS.	+

Abbreviations. ↓: Decrement ↑: Increment EGCG: epigallocatechin-3-gallate; POPC: 1-palmitoyl-2-oleoyl-glycero-3-phosphocholine; TBLE: total brain lipid extract; GST: glutathione S-transferase; PoliQ: polyglutamine; HD: Hungtinton disease; GCG: gallocatechin 3-gallate; GC: gallocatechin; EGC: epigallocatechin; GA: Glatiramer acetate; RRMS: relapsing-remitting multiple sclerosis; EDSS: Expanded Disability Status Scale; MS: multiple sclerosis; MRI: magnetic resonance imaging; AE: adverse event; SAE: several adverse event; PBF: parenchymal brain function; CIS: clinically isolated syndrome; SPMS: secondary progressive multiple sclerosis; EAE: experimental autoimmune encephalomyelitis; NADPH: nicotinamide adenine dinucleotide phosphate; NADH: nicotinamide adenine dinucleotide; NOX: nitrogen oxides; BMI: body mass index; EE: energy expenditure; FAOx: fat oxidation; CHOx: carbohydrate oxidation; PhI: Phase I; PhII: Phase II; NAA: N-acetyl aspartate; IHC: immunohistochemistry; CNS: central nervous system; PCR: polymerase chain reaction; PBS: phosphate-buffered saline; LDH: Lactate dehydrogenase; MOG: myelin oligodendrocyte glycoprotein; PLP: proteolipid protein; Olig 1: oligodendrocyte transcription factor 1; Th 17: T helper 17; Th1: T helper 1;ThT: thioflavin T. GTM: Green tea containing medium. CM: control medium. ↑: increment; ↓: reduction. Quality of evidence grades: high (++++), moderate (+++), low (++), very low (+).

**Table 2:** Catechins in fetal alcohol spectrum disorders.

Author (Year)	Objective	Type of Study and Sample Size	Inclusion and Exclusion Criteria	Interventions	Methodology	Main Outcomes	Conclusion	Quality of Evidence
**Antonio and Druse, 2008** [78]	To investigate the protective effect of EGCG against ethanol-associated apoptosis in rhombencephalic neurons	In-vitro model of rhombencephalic neurons in culture	Culture of rhombencephalic tissue from GD 14 rat fetuses	24-h ethanol treatment of rhombencephalic neurons with 75 mM ethanol. Co-treatment with 75 mM ethanol and 1 µM EGCG	TUNEL for detection and quantification of apoptotic nuclei	Ethanol ↑ apoptosis in fetal rhombencephalic neurons (*p* < 0.01). EGCG ↓ apoptotic rhombencephalic neurons (*p* < 0.01)	The treatment with EGCG provides neuroprotection to the ethanol-treated neurons	+
**Long et al., 2010** [79]	To evaluate the role of oxidative stress in FASD and the effect of EGCG in the prevention of ethanol-induced embryonic damage	Mouse model. Five experimental groups (*n* = 6 per group)	C57BL/6J pregnant female mice. Five experimental groups: (1) control, (2) ethanol 0.005 mL/g, (3) ethanol 0.01 mL/g, (4) ethanol 0.015 mL/g, (5) ethanol 0.02 mL/g +/−EGCG	Ethanol ip (25%, 0.005–0.02 mL/g) on GD8. EGCG intragastric gavage (200, 300 or 400 mg/Kg/day) on GD7–8 in 0.02 mL/g ethanol exposed group	Morphology assessment (GD10.25): HL, HW and CRL. Analysis of neural marker genes and proteins (GD9.25) by RT-PCR and western blot Study of neural marker genes and proteins. Measure H_2_O_2_ and MDA on GD9.25	Ethanol ↑ growth restriction (HL, HW, and CRL). Ethanol ↓ Otx1 and Sox2. Ethanol ↑ H_2_O_2_ and MDA. EGCG ↓ embryo growth restriction EGCG ↑ Otx1 and Sox2	Embryo growth restriction in FASD is mediated by overproduction of ROS. Ethanol affects neural marker genes and proteins involved in brain development and neural differentiation. EGCG has a protective effect against FASD	+
**Tiwari et al., 2010** [80]	To assess the effect of EGCG on ethanol-induced biochemical alterations and behavioral disorders	Rat model. Five experimental groups (*n* = 5–8 per group).	Wistar male rat pups (five-day-old neonates). Five experimental groups: (1) control, (2) ethanol administration, (3) ethanol + EGCG administration (50 mg/Kg), (4) ethanol + EGCG administration (100 mg/Kg), (5) EGCG administration (100 mg/Kg)	Randomization of the pups into five experimental groups. Ethanol administration (5 g/Kg, 12% *v*/*v*) by intragastric gavage. EGCG (50 and 100 mg/kg).	Behavioral tests: MWM test, memory consolidation test, elevated plus maze task. BAC quantification. Acetylcholinesterase activity measurement. Lipid peroxidation, glutathione, superoxide dismutase, catalase and nitrite, TNF-α and IL-1β estimation by ELISA. NF-κB p65 unit quantification. Caspase-3 colorimetric assay	Ethanol ↓ scores in MWM elevated plus maze task. Ethanol ↑ acetylcholinesterase activity, oxidative-nitrosative stress, cytokines (TNF-α and IL-1β), NF- κB and caspase-3 levels. EGCG ↓ behavioral and biochemical ethanol-induced alterations	Cognitive disorders in FASD are associated with oxidative-nitrosative stress-mediated apoptotic signaling. EGCG is useful in the prevention of FASD-related cognitive impairment	++
**Almeida et a., 2021** [81]	To analyze the effects of PAE according two human drinking patterns (Mediterranean vs. binge) on placenta and brain development and evaluate the effect of EGCG treatment on FASD development	Mouse model. Six experimental groups (*n* = 6 per group)	C57BL/6 pregnant mice. Six experimental groups: (1) control med, (2) EtOH med, (3) EtOH med + EGCG, (4) control bin, (5) EtOH bin, (6) EtOH bin + EGCG	Ethanol administration by intragastric gavage from GD0 to GD19, 10% *v*/*v*, 1.5 g/Kg/day or 3 g/Kg/day. EGCG 30 mg/Kg/day by intragastric gavage. Cesarean section at GD19	Fetal and placental weights. Western blot (VEGF-A, PLGF, VEGF-R1, Nrf2, NeuN, DCX, GFAP and BDNF). Immunohistochemistry (VEGF-A, PLGF, VEGF-R1, NeuN, DCX and BDNF). Immunofluorescence (Nrf2 and GFAP)	Ethanol ↓ fetal growth (growth was negatively correlated with ethanol dose). Ethanol ↓ VEGF-A and ↓ VEGF-R1. Ethanol ↓ Nrf2. Ethanol ↓ NeuN, ↑ DCX and ↓ GFAP. EGCG ↓ ethanol-induced oxidative stress ameliorating FASD manifestations	Any drinking pattern may produce fetal growth, loss of mature neurons, delay in neuronal maturation and disorders in astrocyte differentiation (the highest doses cause to the most severe disturbances) EGCG ameliorates FASD alterations	+

Abbreviations: EGCG: epigallocatechin-3-gallate; GD: gestational day; TUNEL: terminal deoxynucleotidyl transferase-mediated dUTP-biotin nick end-labeling; HL: head length; HW: head width; CRL: crown rump length; Oxt1: orthodenticle homolog 1 (Drosophila); Sox2: sex determining region of Y chromosome (Sry); H_2_O_2_: hydrogen peroxide; MDA: malondialdehyde; ROS: radical oxygen species; PND: postnatal days; MWM: Morris water maze; BAC: blood alcohol concentration; TNF: tumor necrosis factor; IL: interleukin; NF- κB: nuclear factor kappa-light-chain-enhancer of activated B cells; FASD: fetal alcohol spectrum disorders; PAE: prenatal alcohol exposure; Med: Mediterranean; Bin: binge; EtOH: ethanol; VEGF-A: vascular endothelial growth factor A; PLGF: placental growth factor; VEGF-R1: vascular endothelial growth factor receptor 1; Nrf2: nuclear factor erythroid 2-related factor 2; NeuN: neuronal nuclei; DXC: doublecortin; GFAP: glial fibrillary acidic protein; BDNF: brain-derived neurotrophic factor. ↑: increment; ↓: reduction. Quality of evidence grades: moderate (+++), low (++), very low (+).

**Table 3:** Neurologic effects of catechins on Down syndrome.

Author (Year)	Objective	Type of Study and Sample Size	Interventions	Methodology	Main Outcomes	Conclusions	Quality of Evidence
**Gu et al., 2020** [50]	To evaluate the effect of EGCG on DYRK1A kinase activity	C57Bl/6J mice overexpressing Dyrk1A (TgBACDyrk1A) model (*n* = 30) (*n* = 30)	FontUp administration by oral gavage (25 mg/kg, 50 mg/kg or 75 mg/kg).	Three experimental groups (FontUp administration of 25 mg/kg, 50 mg/kg, or 75 mg/kg) Protein extraction and analysis. Truncated DYRK1A (DYRK1A-ΔC) purification. DYRK1A inhibition analysis HPLC analysis for FontUp polyphenols quantification. Computational molecular docking (ECG) and epicatechin (EC) on DYRK1A kinase activity.	EGCG and ECG ↓ DYRK1A activity EGC and EC = DYRK1A activity. FontUp = liver and cardiac function EGCG crosses blood-brain barrier	Oral FontUp^®^ normalized brain and plasma biomarkers altered in TgBACDyrk1A, without damaging liver and cardiac performances	++
**Catuara-Solarz et al., 2016** [96]	To explore the effects of a combined therapy with EE and EGCG on neurological disorders of DS at young age	Experimental Ts65Dn 1–2-month-female mouse models of DS. (WT = 8; TS = 7; WT-EE-EGCG = 7; TS-EE-EGCG = 7)	Green tea extract containing 45% EGCG administrations by oral feeding (EGCG dosage: 0.326 mg/mL, 0.65 mg per day; 30 mg/kg per day during 30 days)	Four experimental groups (WT, TS, WT-EE-EGCG, TS-EE-EGCG). Morris Water Maze Novel object recognition test Quantification of dendritic spine density. Immunohistochemistry for synaptic studies Immunohistochemistry for synaptic modifications studies	EE-EGCG treatment ↑ corticohippocampal-dependent learning and memory. ↑ cornu ammonis 1 (CA1) dendritic spine density. Improvement of excitatory and inhibitory synaptic markers in CA1 and dentate gyrus	EE-EGCG treatment derived cognitive improvements are linked to modulation of neural synapsis at the hippocampus and normalization in dendritic spine density	++
**Goodlett et al., 2020** [97]	To test the effects of EGCG on neurobehavioral and skeletal phenotypes in a mouse model	Experimental Ts65Dn mouse models of DS (Euploid PB = 13 Euploid EGCG = 11 Ts65Dn PB = 12 Ts65Dn EGCG = 8	Three-week EGCG oral gavage therapy (200 mg/kg/day)	Multivariate concentric square field maze. BB and MWM. Morris water maze CT imaging HPLC kinase activity assay WB for DYIRK1A protein quantification	↓ growth in both euploid and trisomic mice =results in on conductual assessment of Ts65Dn mice. Ts65Dn mice. ↓ cortical bone formation and potency in Ts65Dn mice	EGCG has no effects on behavior. High-dose EGCG caused deleterious effects on growth and skeletal phenotypes	++
**Stringer et al., 2015** [98]	To evaluate the therapeutic effects of EGCG on locomotor activity and learning and memory on a mice model of DS	Experimental Ts65Dn mouse models of DS and euploid treatments: Ts65Dn—EGCG *n* = 8, water *n* = 9; Euploid—EGCG *n* = 12, water *n* = 13	Ts65Dn or euploid mouse models were randomized to receive EGCG + H3PO4 (*n* = 10/*n* = 8), EGCG (*n* = 14/*n* = 8), water + H3PO4 (*n* = 9/*n* = 13) or water (*n* = 9/*n* = 17) for three months	20 mg/kg/day EGCG HPLC/MS degradation analysis Locomotor activity assessment, NOR, DNMP, BB and MWM. Dyrk1A kinase activity assay	Ts65Dn ↑ locomotive performance Ts65Dn↓ novel object detection, balance beam and spatial learning and memory. EGCG did not ameliorate performance of the Ts65Dn mice on these tasks	Oral EGCG treatment up to 20 mg/kg/day did not improve learning and memory performance in adolescent Ts65Dn mice	++
**Souchet et al., 2015** [95]	To investigate the consequences of one-month therapy with EGCG-containing products on excitation/inhibition balance in DS adults.	Experimental adult mBACtgDyrk1a mice Transgenic (TG) = 10 Wild-type (WT) = 10 Treated transgenic (TG*) = 10	Administration of 225 mg/kg/day of Polyphenon 60 for four months in adult mice mBACtgDyrk1a or for six weeks before and during behavioral analysis in Ts65Dn	Indicators of GABAergic and glutaminergic synaptic routes were evaluated by immunoblot Y-maze paradigm to assess working memory	↓ GABA in cortex, hippocampus and cerebellum ↑ GLUR1, NR1, NR2a, VGLUT1 in cortex ↑ Ratio of PCAMKII/CAMKII in the hippocampus ↑ Short term memory	EGCG therapy restores excitation/inhibition balance disorders in DS adults	++
**Stringer et al., 2017** [99]	To investigate if an EGCG would yield improvements in either cognitive or skeletal deficits.	Experimental Ts65Dn mouse models of DS Eup + water = 19 Eup + EGCG = 18 Tsg + water = 13 Tsg + EGCG = 15	Oral administration of EGCG from PD24 to PD68	MCSF, NOR, BB, and MWM. CT, HPLC kinase activity assay and WB for Dyrk1A quantification	=growth ↓ Kinase activity (cerebellum) =cognitive deficits ↑ Adverse changes in skeleton	No beneficial therapeutic effects were seen with EGCG intake on behavior. Caused detrimental skeletal effects in Ts65Dn mice.	++
**Catuara et al., 2015** [100]	To investigate the effect of coadjuvant treatment with EGCG and EE on the cognitive decline in DS.	Experimental Ts65Dn mouse models of DS 5–6 months old female mice WT = 10; TS = 11; WT-EE = 14; TS-EE = 11; WT-EGCG = 11; TS-EGCG = 9; WT-EE-EGCG = 12; TS-EE-EGCG = 8.	Administration of green tea extract including 45% EGCG (0.326 mg/mL, 0.9 mg per day; 30 mg/kg per day) during 30 days	MWM for hippocampal-dependent learning and memory evaluation.	EGCG or EE = spatial learning EGCG + EE ↓ learning alterations of middle age Ts65Dn mice and this stratification continued upon treatments	Combining EE and EGCG ameliorates age-related cognitive degeneration in DS	++
**Valenti 2013 Italy** [101]	To test the capability of EGCG to reestablish the energy in mitochondria and reduce oxidative stress in DS cells	Experimental Cultured lymphoblasts and fibroblasts from DS patients	Lymphoblastoid and fibroblast cells were treated with 20 μM EGCG joined to the cell culture for 24 h	Assessment of mitochondrial ATP production rate, cellular ATP and ROS detection	↑ mitochondrial complex I and ATP synthase catalytic action ↓ oxidative stress. ↑ mitochondrial biogenesis ↑ Sirt1-dependent PGC-1α deacetylation, NRF-1 and T-FAM protein levels	EGCG antioxidant effects rescues mitochondrial energy and ROS production impairment, prevents overproduction of reactive oxygen species (ROS) and peroxidation of lipid membranes and increases mitochondrial biogenesis	++
**Valenti et al., 2016** [102]	To establish possible function of mitochondria in DS cognitive disability	Experimental Ts65Dn mouse models of DS Ts65Dn = 12 WT = 12	NPCs, cultured for 48 h were complemented with EGCG and RSV, at a concentration of 20 μM and 10 μM, respectively	Measurements of oxygen consumption, mitochondrial ATP production and L-lactate, mitochondrial chain complex activities, ROS production. Immunoblot analysis, quantitative analysis of mtDNA content.	↑ Oxidative phosphorylation ↑ Mitochondrial biogenesis ↑ Proliferation of NCPs ↑ PGC-1α/Sirt1/AMK axis mitochondrial energy, and improved growth of NPCs	EGCG and RSV reactivates mitochondria bioenergetics and biogenesis and promotes NPCs in DS	++
**Stagni et al., 2016** [103]	To study the effect of EGCG in hippocampal development and memory performance	Experimental Ts65Dn mouse models of DS EGCG: Euploid *n* = 40 and Ts65Dn *n* = 25 NT:Age-matched euploid (*n* = 53) and Ts65Dn (*n* = 25)	EGCG daily subcutaneous injection from PN3 to PN15 (25 mg/kg).	Nissl staining Immunohistochemistry (ki67, cleaved caspase-3, BrdU, NeuN, GFAP, Ayn, PSD-95) Western Blot (GSK3β) MWM and Y-maze	=brain and body weight =locomotor activity and learning and memory Short term effects: ↑ Ki67, BrdU, granular cells in hippocampus, SYN, PSD-95 ↓ GSK3β Long term effects: ↓ BrdU/NeuN, BrdU/GFAP, GSK3β =Ki67, SYN, PSD-95	EGCG rescues hippocampal neurogenesis and synaptic processes but these effects do not persist for a long time.	++
**de La Torre et al., 2014** [104]	To explore if EGCG rescues the intellectual disabilities in adult DS	-Experimental Ts65Dn or TgDyrk1A mouse models of DS: WT untreated *n* = 13/*n* = 19; TG/Ts65Dn–untreated *n* = 16/*n* = 14; WT-EGCG *n* = 16/*n* = 22; TG-EGCG/Ts65Dn *n* = 14/*n* = 14.14. -A randomized, double blind, placebo-controlled study in DS humans: 13 EGCG group 16 placebo group	Mice were administered EGCG in drinking water for one month (90 mg/mL for a dose of 2–3 mg per day) Pilot study: groups were randomized to receive oral EGCG at dosage of 9 mg/kg/day or placebo over six months	Mouse model: -Water maze for hippocampal-dependent spatial recognition -NOR for learning and memory assessment deficits. -Dyrk1A kinase activity and homocysteine evaluation Pilot study: -Neurophysiological testing -Blood test and ALT, AST, glucose, cholesterol, TG, GSH-Px analysis -HPLC/MS for EGCG determinations	Mouse model: ↓ DYRK1A ↑ homocysteine ↑ Learning and memory Pilot study: =ALT, AST, TG, glucose, GSH-Px ↓ cholesterol ↑ Episodic and learning memory, visual memory recognition	EGCG improved learning and memory disorders in DS, blocking Dyrk1A expression Plasmatic homocysteine are a biomarker of hippocampal DYRK1A activity in human study	++++
**de La Torre et al., 2016** [85]	To test if the administration of EGCG would enhance the outcomes of intellectual rehabilitation in young adults with DS	Double blind, placebo-controlled, phase 2, single center trial (TESDAD) in DS humans 43: EGCG and intellectual treatment group 41: placebo and intellectual treatment group Aged 16–34 years	Randomization and EGCG (9 mg/kg per day) or placebo and cognitive rehabilitation for 12 months. Follow- up of 6 months after intervention discontinuation	Intellectual assessment for working memory, executive performance. Homocysteine, Dyrk1A kinase activity, ALT, AST, cholesterol, TG measurements. fMRI and TMS for functional connectivity patterns studies.	↓ Cholesterol ↑ Homocysteine ↑ Inhibitory control, recognition memory, adaptive behavior =BMI ↑ Brain connectivity	EGCG joined to intellectual rehabilitation for 12 months had greater results than placebo and intellectual rehabilitation at improving visual memorial perception conduct control, and compliant behavior	++++
**De Toma et al., 2019** [105]	To compare proteomic changes in DS EE or GTE treated DS individuals	Experimental Ts65Dn male mouse models of DS at thee months 144 animals: 38 NT mice (18 TG, 20 WT); 38 EGCG (18 TG, 21 WT); 36 EE (16 TG, 18 WT); 33 EGCG + EE (16 TG, 17 WT)	EGCG 326.25 mg per capsule mixed with drinking water at 0.33 mg/mL at medium dose of 42 mg/kg per day for one month.	Western Blot for Dyrk1A quantification Mass-spectrometry-based proteomics Liquid chromatography tandem-mass spectrometry NOR	-Dyrk1A overexpression impacted the phosphoproteome in TG hippocampus (mainly proteins plasticity and cognitive-related proteins) -These (phospho-) proteomic changes were rescued by green tea and/or EE	-t Dyrk1A overexpression causes changes in the proteome and phosphoproteome of the hippocampus of transgenic mice -The cognitive enhancer treatments rescued these alterations	++
**De Toma et al., 2020** [106]	To study the effects of EGCG, EE and their mixture using proteome, and phosphoproteome analysis in the hippocampi of DS	Experimental Ts65Dn mouse models of DS Five mice per group, randomly chosen (40 total mice)	EGCG: 326.25 mg per capsule. mixed with water at 0.33 mg/mL equivalent to a medium dose of 42 mg/kg per day for one month.	Mass-spectrometry-based proteomics Liquid chromatography tandem-mass spectrometry Western blot.	-Neurocognitive-related GTPase/kinase activity and chromatin proteins were impaired. -EGCG, EE, and their mixture rescued higher than 70% of the phosphoprotein impairment in Ts65Dn, and induced probable beneficial effects	Green tea extracts may restore an appropriate epigenetic profile and reverse the kinome deregulation promoting the cognitive rescue	++
**Starbuck et al., 2021** [107]	To investigate the effect of GTE-EGCG for ameliorating facial dysmorphologies associated with DS	Experimental mouse models 55 Ts65Dn Cross sectional study in children 0–18 years old with DS 63 DS 4 mosaics 13 treated with EGCG 207 euploids	High (100 mg/kg/day) or low doses (30 mg/kg/day) of GTE-EGCG, were administered from embryonic Day 9 to Day 29 post-delivery, in mouse models. Children with DS received low doses of EGCG	Morphometric facial analysis evaluation by CT 3D quantitative morphometric measures of the face in mice and photogrammetry in humans	-The smallest GTE-EGCG dose ameliorated facial skeleton characteristics in a mouse model of DS. -In humans, GTE-EGCG administration restored facial dysmorphic features in children with DS if therapy was given over the first 3 years of life. -Greater GTE-EGCG dosing disrupted normal growth and augmented facial dysmorphic features in trisomy and euploid mice	GTE-EGCG modulates facial development with dose-dependent effects, but high doses have potentially detrimental effects observed in mice	+++

Abbreviations: EGCG: Epigallocatechin-gallate; DS: Down syndrome; HPLC: high performance liquid chromatography; GTE: green tea extract; EE: environmental enrichment; MWM: Morris water maze spatial learning task; NOR: novel object recognition; BB: balance beam task; WT: wild type mouse; NT: no treatment; CT: computerized tomography; NOR: novel object recognition; DNMP: T-maze delayed non-matching to place; MCSF: multivariate concentric square field; NPCs: neural precursor cells; NRF-1: nuclear respiratory factor; T-FAM: mitochondrial transcription factor; RSV: resveratrol; PN: postnatal; BrdU: bromodeoxyuridine; SYN: synaptophisin; PSD-95: post-synaptic density protein; GSK3β: glycogen synthase kinase 3β; NeuN: neuronal nuclei; GFAP: glial fibrillary acidic protein; ALT: alanine transaminase; AST aspartate transaminase; TG: triglycerides; GSH-Px: glutathione peroxidase; BMI: body mass index; fMRI: functional magnetic resonance imaging; TMS: Transcranial magnetic stimulation. ↑: increment; ↓: reduction. Quality of evidence grades: High (++++), moderate (+++), low (++), very low (+).

**Table 4:** Neurologic effect of catechins on healthy populations.

Author (Year)	Objective	Type of Study and Sample Size	Inclusion and Exclusion Criteria	Interventions	Methodology	Main Outcomes	Conclusion	Quality of Evidence
**Kesse-Guyot et al., 2012** [108]	To evaluate the association between polyphenol intake and cognitive function, 13 years later	Prospective Observational *n* = 2574	45–60 years old	-	24 h dietary records every two months for two years. Evaluation of episodic memory, lexical-semantic memory, working memory and mental flexibility	Catechins ↑ scores on language and verbal memory score, and ↓ scores on executive functioning factor	Catechins may be associated with dual effects on the brain	++
**Biasibetti et al., 2013** [109]	To explore the association between flavonoidintake and cognitive health	Observational. *n* = 808	Inclusion: Individuals living on Catania, Italy. >50 years old. Exclusion: FFQ with unreliable intakes	-	Administration of two FFQ	Higher dietary intake of catechins ↓ impaired cognitive status. (Q4 vs. Q1: OR = 0.24, 95% CI: 0.08, 0.72)	Individuals with higher flavonoid intake may have better cognitive health	++
**Mohamed et al., 2013** [114]	To evaluate the cognitive effects of OPLE in humans and rats	Human: placebo -controlled trial. *n* = 15. Animal (*n* = 8): normal rat groups: control, OPLE, captopril. NO-deficient rat groups: vehicle, OPLE, captopril	Young, healthy, adult human volunteers. Rats: 16-week-old male Wistar Kyoto	Human: 500 mg OPLE/day or placebo. Rat: 500 mg OPLE/kg day or L-NAME (60 mg/L), captopril (100 mg/kg day)	Human: Cognitive tests at 0, 1, and 2 months evaluating short-term memory, processing speed, spatial visualization ability, and language skills. Rat: evaluation of hippocampal neuron damage. Evaluation of SOD activity	OPLE: ↑ Short term memory, ↑ spatial visualization ability, ↑ processing speed. OPLE ↑ hippocampal pyramidal and granule viable cells in NO-deficient rats	OPLE showed neuroprotection in NO-deficient rats, and a cognitive amelioration in humans	++
**Scholey et al., 2012** [110]	To investigate if EGCG modulates brain activity and self-reported mood	Double blind, placebo controlled crossover study. *n* = 31	Inclusion: normal BMI, right-handed, no drugs or natural therapies, English speaking, healthy population	Administration of Teavigo (94% EGCG) 300 mg	Mood and an EEG before and 120 min after EGCG administration or placebo	EGCG ↑ alpha, beta and theta activity on the EEG, ↑ self-rated calmness, ↓ self-rated stress	EGCG group may have been in a morerelaxed and attentive state	+++
**Emma et al., 2012** [111]	To elucidate the effects of EGCG on cognitive performance, mood and CBF in humans	Double blind, placebo-controlled, crossover study. *n* = 27	Exclusion: drugs and herbal extracts/food supplements, head injury, neurological disorder, allergies or intolerances, more than 600 mg/day of caffeine	Administration of placebo and EGCG at two doses (135 and 270 mg)	After 45 min, performed cognitive tasks and measured the CBF and hemodynamics in the frontal cortex using near-infrared spectroscopy	No differences on cognitive performance and mood with EGCG use. EGCG 135 mg ↓ CBF in the frontal cortex. No differences with 270 mg EGCG	EGCG 135 mg modulated CBF	+++
**Dietz et al., 2017** [113]	To investigate the effects of matcha on mood and cognitive performance	Randomized, placebo-controlled, single blind, crossover study. *n* = 27	Inclusion: 100–400 mg caffeine per day. Exclusion: health problems, pregnancy, lactation, allergies/intolerances, drugs, night shift work, abnormal sleep pattern	Administration of matcha tea or bar with 4.0 g of matcha tea (67 mg L-theanine, 280 mg EGCG, 136 mg caffeine) or placebo	Preformation of CDR test battery and POMS test before and 60 min after administration	With matcha products ↑ basic attention abilities, ↑ psychomotor speed response. No mood changes	Matcha tea induce slight effects on episodic secondary memory and speed of attention	++
**Liu et al., 2018** [112]	To evaluate if green tea extract affects working memory in young and older women	Single blind, placebo-controlled, crossover design. *n* = 20	Inclusion: Age: 18–30 or 50–70, Caucasian, healthy, normal BMI, non-smoking, waist circumference < 35 inches. Exclusion: pregnancy or lactating, use of green tea products, medications, laboratory test alterations	Decaffeinated GTE 5.4g and placebo, one-week washout period	Working memory tests. Blood extraction and serum analysis of MDA concentration and total TEAC	GTE ↑ reading span performance in older women, no differences on younger group. No differences on plasma concentration of TEAC and MDA	Acute administration of GTE may improve 50–63 aged woman working memory	++
**Ide et al., 2016** [5]	To examine if green tea intake modifies cognitive dysfunction	Double blind, randomized controlled study. *n*= 33	Inclusion: age: ≥ 50, ability to orally ingest powders, no supplements, MMSE-J score < 28. Exclusion: tea allergy, severe diseases	Consumption of 2.0 g/day of green tea (220.2 mg of catechins) or placebo powder for one year	MMSE-J performance every 3 months. Laboratory tests	No changes of MMSE-J score after o ne year comparted with placebo. Green tea group: ↓ MDA-modified low-density lipoprotein	Green tea consumption may not affect cognitive function but prevent oxidative stress increase in elderly population	++++
**Baba et al., 2020** [115]	To investigate the effect of decaffeinated GTC on cognitive function	Double blind, randomized, controlled study. *n* = 52	Inclusion: age: 50–69, self-assessed cognitive decline, MMSE-J score > 24, non-smokers. Exclusion: serious diseases, medication or supplements, extremely unbalanced diets, irregular habits, psychiatric problems or alcoholism	Placebo or GTC capsules (336.4 mg) per day for 12 weeks	MMSE-J and blood biomarkers measured initially and 12 weeks after ingestion. Cognitrax test battery before starting, after first dose, and after 12 weeks of daily administration	Catechin group after 12 weeks: ↑ Working Memory Tasks. No differences on memory tasks, Facial Expression Recognition Tasks, Visual Information Processing Tasks, Motor Function Tasks and blood Biomarkers	GTC may improve working memory	++++
**Sakurai et al., 2020** [116]	To evaluate if matcha tea extract affect cognitive function or impulsivity in the elderly	Randomized, double blind, placebo-controlled trial, *n* = 54	Inclusion: age: < 60. No diagnosis of dementia and MCI	3 g Matcha or placebo powder per day, for 12 weeks	Evaluation of cognitive (MoCA and MMSE) and memory function tests (WMS-DR) and assessment of Impulsivity (BIS-11)	No changes on cognitive test, memory and impulsivity scores. On the women subgroup ↑ MoCA in the matcha group	Daily Matcha Green Tea consumption may have protect against cognitive decline in elderly women	++++
**Unno et al., 2020** [117]	To examine which GTCdose is necessary to prevent age-related cognitive decline	Experimental animal. *n* = 180 mice/six groups	Four-week-old male SAMP10 mice	Oral administration of GTC intake of 0, 1, 5, 15, 30, 60 mg/kg depending on the group	Memory and working memory tests. Euthanasia at 2, 6, 12 month and prefrontal cortex, hippocampus and blood samples. Exam of qRT-PCR evaluating Egr2, Nr4a1, Fos, Arc, Egr1, Npas4, and Cyr61 genes	↑ Long-term memory on the group with 60 mg/kg of GTC. ↑ Working memory on the groups with 30 and 60mg/kg. ↑Nr4a, Fos, Egr1, Npas4 and Cyr61	GTC suppressed age-related cognitive decline	+
**Ramis et al., 2020** [118]	To study the effect of GTE on brain and cognitive status of old rats	Experimental animal. *n* = 16	Old male rats (18 months)	20 mg/kg/day of IP poliphenon-60 or catechin or vehicle for 28 days	Behavioral tests. Exam of the activity of TPH and TH. Euthanasia and Western Blot Analysis	↑ working memory and episodic memory. ↑ noradrenergic, serotonergic and dopaminergic system. ↓ age associated neuroinflammation by modulating SIRT	Consumption of green tea delays cerebral senesce on rats	+

Abbreviations: ↓: Decrement ↑: Increment, FFQ: frequency questionnaires, Q: quartile, OPLE: alcoholic extracts of oil palm leaves, NO: nitride oxide, L-NAME: N omega-Nitro-L-arginine methyl ester hydrochloride, SOD: superoxide dismutase, EGCG: Epigallocatechin-gallate, BMI: body mass index, EEG: electroencephalogram, CBF: cerebral blood flow, CDR: cognitive Drug Research, POMS: Profile of Mood States, GTE: green tea extract, MDA: malondialdehyde, TEAC: total antioxidant capacity, MMSE-J Mini-Mental State Examination Japanese version, GTC: green tea catechin, MCI: mild cognitive impairment), MoCA: Montreal cognitive assessment, WMS_DR: Wechsler Memory Scale-Delayed Recall, BIS-11: Barratt Impulsiveness Scale-11, SAMP10: senescence-accelerated mouse prone 10/TaSlc, qRT-PCR: quantitative real time polymerase chain reaction, Egr2: early growth response 2, Arc: activity regulated cytoskeletal-associated protein, Nr4a1: nuclear receptor subfamily 4, Fos: FBJ osteosarcoma oncogene, Egr1:early growth response 1, Npas4: neuronal PAS domain protein 4, Cyr61: cysteine-rich protein 61, IP: intraperitoneally, TPH: tryptophan hydroxylase, TH: tyrosine hydroxylase, SIRT1: protein sirtuin 1, ↑: increment, ↓: reduction Quality of evidence grades: high (++++), moderate (+++), low (++), very low (+).

## Supplementary Materials

Available online at https://www.mdpi.com/article/10.3390/nu13072232/s1. Supplemental Material S1: Search Methodology. Table S1: Summary of the Grading of Recommendation, Assessment, Development, and Evaluation approach (GRADE) evidence profile for neurodegenerative diseases. Table S2: Summary of GRADE evidence profile for fetal alcohol spectrum disorders. Table S3: Summary of GRADE evidence profile for Down syndrome. Table S4: Summary of GRADE evidence profile on the neurologic effects of catechins in healthy populations.
