# Supplementary material for: Therapeutic Effects of Catechins in Less Common Neurological and Neurodegenerative Disorders
Source: Nutrients. 2021 Jun 29;13(7):2232. doi: 10.3390/nu13072232 (PMC8308206; doi:10.3390/nu13072232)
Supplement: Supplementary file 1 [file nutrients-13-02232-s001.zip › nutrients-1216442-supplementary.pdf]

Supplemental Material S1

Search methodology

Section 3.1. Search: ((("catechin"[MeSH Terms] OR "catechin"[All Fields] OR "catechins"[All Fields] OR "catechine"[All Fields] OR "catechines"[All Fields]) NOT ("polyphenol s"[All Fields] OR "polyphenoles"[All Fields] OR "polyphenolic"[All Fields] OR "polyphenolics"[All Fields] OR "polyphenols"[MeSH Terms] OR "polyphenols"[All Fields] OR "polyphenol"[All Fields])) AND ("antioxidant s"[All Fields] OR "antioxidants"[Pharmacological Action] OR "antioxidants"[MeSH Terms] OR "antioxidants"[All Fields] OR "antioxidant"[All Fields] OR "antioxidating"[All Fields] OR "antioxidation"[All Fields] OR "antioxidative"[All Fields] OR "antioxidatively"[All Fields] OR "antioxidatives"[All Fields] OR "antioxidizing"[All Fields] OR ("anti inflammatory agents"[Pharmacological Action] OR "anti inflammatory agents"[MeSH Terms] OR ("anti inflammatory"[All Fields] AND "agents"[All Fields]) OR "anti inflammatory agents"[All Fields] OR "antiinflammatories"[All Fields] OR "antiinflammatory"[All Fields])) AND ("neurodegenerative"[All Fields] OR "neurodegeneratives"[All Fields])) AND (2010:2021[pdat]). Filters: Article types: classical article, clinical study, clinical trial, comparative study, controlled clinical trial, journal article, multicenter study, observational study, randomized con-trolled trial. Publication dates: 2010-2021(included); Species: humans, mouse, rat; Languages: English.

Search: ("autophagies"[All Fields] OR "autophagy"[MeSH Terms] OR "autophagy"[All Fields] OR "autophagy s"[All Fields]) AND ("catechin"[MeSH Terms] OR "catechin"[All Fields] OR "catechins"[All Fields] OR "catechine"[All Fields] OR "catechines"[All Fields]) AND ("neuron s"[All Fields] OR "neuronal"[All Fields] OR "neuronally"[All Fields] OR "neuronals"[All Fields] OR "neurone s"[All Fields] OR "neurones"[All Fields] OR "neuronic"[All Fields] OR "neurons"[MeSH Terms] OR "neurons"[All Fields] OR "neuron"[All Fields] OR "neurone"[All Fields] OR ("neurodegenerative"[All Fields] OR "neurodegeneratives"[All Fields])). Filters: Article types: classical article, clinical study, clinical trial, comparative study, controlled clinical trial, journal article, multicenter study, observational study, randomized con-trolled trial. Publication dates: 2010-2021(included); Species: humans, mouse, rat; Languages: English.

Search: ("autophagies"[All Fields] OR "autophagy"[MeSH Terms] OR "autophagy"[All Fields] OR "autophagy s"[All Fields]) AND ("catechin"[MeSH Terms] OR "catechin"[All Fields] OR "catechins"[All Fields] OR "catechine"[All Fields] OR "catechines"[All Fields]) AND ("neuron s"[All Fields] OR "neuronal"[All Fields] OR "neuronally"[All Fields] OR "neuronals"[All Fields] OR "neurone s"[All Fields] OR "neurones"[All Fields] OR "neuronic"[All Fields] OR "neurons"[MeSH Terms] OR "neurons"[All Fields] OR "neuron"[All Fields] OR "neurone"[All Fields] OR ("neurodegenerative"[All Fields] OR "neurodegeneratives"[All Fields])). Filters: Article types: classical article, clinical study, clinical trial, comparative study, controlled clinical trial, journal article, multicenter study, observational study, randomized con-trolled trial. Publication dates: 2010-2021(included); Species: humans, mouse, rat; Languages: English.

Search: ("catechin"[MeSH Terms] OR "catechin"[All Fields] OR "catechins"[All Fields] OR "catechine"[All Fields] OR "catechines"[All Fields]) AND "DYRK1A"[All Fields]. Filters: Article types: classical article, clinical study, clinical trial, comparative study, controlled clinical trial, journal article, multicenter study, observational study, randomized con-trolled trial. Publication dates: 2010-2021(included); Species: humans, mouse, rat; Languages: English.

Section 3.2. Search: ("epigallocatechin gallate"[Supplementary Concept] OR "epigallocatechin gallate"[All Fields] OR ("tea"[MeSH Terms] OR "tea"[All Fields] OR ("green"[All Fields] AND "tea"[All Fields]) OR "green tea"[All Fields])) AND ("huntington disease"[MeSH Terms] OR ("huntington"[All Fields] AND "disease"[All Fields]) OR "huntington disease"[All Fields]). Filters: Article types: classical article, clinical study,

clinical trial, comparative study, controlled clinical trial, journal article, multicenter study, observational study, randomized con-trolled trial. Publication dates: 2010-2021(included); Species: humans, mouse, rat; Languages: English.

Search: ("multiple sclerosis"[MeSH Terms] OR ("multiple"[All Fields] AND "sclerosis"[All Fields]) OR "multiple sclerosis"[All Fields]); (catechin: "catechin"[MeSH Terms] OR "catechin"[All Fields] OR "catechins"[All Fields] OR "catechine"[All Fields] OR "catechines"[All Fields]). Filters: Article types: classical article, clinical study, clinical trial, comparative study, controlled clinical trial, journal article, multicenter study, observational study, randomized controlled trial; Publication dates: 2010-2021(included); Species: humans, mouse, rat; Languages: English.

Section 3.3. Search: ("Fetal Alcohol Spectrum Disorders"[Mesh]) AND "Catechin"[Mesh]: 1 result (((("Fetal Alcohol Spectrum Disorders"[Mesh]) OR "Alcohol-Related Disorders"[Mesh]) OR "Alcohol-Induced Disorders"[Mesh]) AND ("Catechin"[Mesh]). ("Fetal Alcohol Spectrum Disorders"[Mesh]) AND "epigallocatechin gallate" [Supplementary Concept]. Filters: Article types: classical article, clinical study, clinical trial, comparative study, controlled clinical trial, journal article, multicenter study, observational study, randomized controlled trial; Publication dates: 2010-2021(included); Species: humans, mice, rat; Languages: English.

Section 3.4. (("epigallocatechin gallate"[Supplementary Concept] OR "epigallocatechin gallate"[All Fields] OR ("tea"[MeSH Terms] OR "tea"[All Fields] OR ("green"[All Fields] AND "tea"[All Fields]) OR "green tea"[All Fields])) AND ("down syndrome"[MeSH Terms] OR ("down"[All Fields] AND "syndrome"[All Fields]) OR "down syndrome"[All Fields])) AND (2010:2021[pdat]). Filters: Article types: classical article, clinical study, clinical trial, comparative study, controlled clinical trial, journal article, multicenter study, observational study, randomized con-trolled trial. Publication dates: 2010-2021(included); Species: humans, mouse, rat; Languages: English.

Section 3.5. Search: ("health"[MeSH Terms] OR "health"[All Fields] OR "health's"[All Fields] OR "healthful"[All Fields] OR "healthfulness"[All Fields] OR "healths"[All Fields]); ("healthies"[All Fields] OR "healthy"[All Fields]); ("catechin"[MeSH Terms] OR "catechin"[All Fields] OR "catechins"[All Fields] OR "catechine"[All Fields] OR "catechines"[All Fields]). ("neurology"[MeSH Terms] OR "neurology"[All Fields] OR "neurology's"[All Fields]); ("mental"[All Fields] OR "mentalities"[All Fields] OR "mentality"[All Fields] OR "mentalization"[MeSH Terms] OR "mentalization"[All Fields] OR "mentalizing"[All Fields] OR "mentalize"[All Fields] OR "mentalized"[All Fields] OR "mentally"[All Fields]); ("cognition"[MeSH Terms] OR "cognition"[All Fields] OR "cognitions"[All Fields] OR "cognitive"[All Fields] OR "cognitively"[All Fields] OR "cognitives"[All Fields]). Filters: Article types: classical article, clinical study, clinical trial, comparative study, controlled clinical trial, journal article, multicenter study, observational study, randomized con-trolled trial. Publication dates: 2010-2021(included); Species: humans; Languages: English.

Supplementary tables

Table S1. Summary of the Grading of Recommendation, Assessment, Development, and Evaluation approach (GRADE) evidence profile for neurodegenerative diseases.

**Quality Assessment**						**No. of Subjects**		**Effect**		**Quality**	**Importance**
**No. of Studies**	**Design**	**Risk of Bias**	**Inconsistency**	**Indirectness**	**Imprecision**	**No. of Patients**	**Control**	**Relative (95% CI)**	**Absolute**		
**Huntington disease**											
**Beasley et al., 2019** [1], **Varga et al., 2018** [2], **Ehrnhoefer et al., 2006** [3]**: Ability of EGCG to modify htt aggregation (no follow-up)**											
**3**	Experimental	No serious risk of bias	No serious inconsistency	No serious indirectness	No serious imprecision	In vitro or Drosophila models		EGCG acted as a potent inhibitor of fibril formation ($p < 0.05$)		Low ++	Important
**Multiple sclerosis**											
**Mähler et al., 2015 [94]: Metabolic response to EGCG in MS (12 weeks of follow-up)**											
**1**	DBPC crossover	No serious risk of bias	No serious inconsistency	Indirectness (subrogated variables)	Serious imprecision (low sample size)	18	18	Working efficiency: placebo: 20 +/−3 EGCG: 25 +/−6 (p = 0.004) Postprandial FAOx: placebo: 8.3 +/−4.3 EGCG: 8.6 +/−5.0 (sex differences)		Low ++	Important
**Mossakowski et al., 2015 [93]: Tracking sources of oxidative stress during chronic neuroinflammation (no follow-up)**											
**1**	RDBPC	No serious risk of bias	No serious inconsistency	Indirectness (subrogated variables)	Serious imprecision	n = 6 RRMS + EGCG + GA	n = 6 RRMS + GA<br>6 RRMS<br>6 CIS<br>6 controls<br>6 SPMS	NOX activity. on CD11b+: Control 3.4 ± 0.6%. RRMS untreated: 18.3 ± 2.5%, RRMS + GA: 10.8 ± 2.8%, RRMS + GA+ EGCG: 6.4 ± 1.2%		Low ++	Less important
**Lovera et al., 2012 [96]: Safety and futileness of Polyphenon in MS (Ph1 and Ph2) (six months of follow-up)**											
**1**	Ph1: single group<br>Ph2: RDBPC	No serious	No serious inconsistency	No serious indirectness	Serious imprecision	Ph1: 10<br>Ph2: 8	Ph2: 5	NAA adjusted for creatinine: 10% [3.4%,16.2%]		Moderate +++	Important

		risk of bias						Ph2 was stopped before because 5/7 participants had elevated liver enzymes			
**Bellmann–Strobl et al., 2021** [4], **Rust et al., 2021** [5]**: Efficacy and safety of EGCG treatment in MS (36 months follow-up)**											
**2**	RDBPC	No serious risk of bias	No serious inconsistency	No serious indirectness	No serious imprecision	92	91	No differences on efficacy. No differences on adverse events.		High ++++	Important

Abbreviations: EGCG: epigallocatechin-3-gallate; DBPC: Double blind placebo controlled study; Rt-PA: recombinant tissue plasminogen activator; NIHSS: National Institutes of Health stroke scale; OTT: onset-to-treatment time; GA: glatiramer acetate; FAOx: fat oxidation; RDBPC: Randomized double blind placebo controlled study; RRMS: relapsing-remitting multiple sclerosis; CIS: clinically isolated syndrome; SPMS: secondary progressive multiple sclerosis; NOX: nicotinamide adenine dinucleotide phosphate (NADPH) oxidases; EAE: experimental autoimmune encephalomyelitis; PhI: Phase I; PhII: Phase II; NAA: N-acetyl aspartate; MS: multiple sclerosis; HD: Huntington disease; HTT: huntingtin. Quality of evidence grades: high (++++), moderate (+++), low (++).

Table S2. Summary of GRADE evidence profile for fetal alcohol spectrum disorders.

**Quality Assessment**						**No. of Patients**		**Effect**		**Quality**	**Importance**
**Nº of studies**	**Design**	**Risk of Bias**	**Inconsistency**	**Indirectness**	**Imprecision**	**Cases**	**Control**	**Relative (95%CI)**	**Absolute**		
**Long (2010)** [6]**, Almeida (2021)** [7]**: EGCG and growth restriction prevention (no follow-up)**											
**2**	Experimental C57BL/6 mouse models of FASD.	No serious risk of bias	Serious inconsistency (assessment of different measures)	No serious indirectness	No serious imprecision	48	18	Fetal growth improvement ($p < 0.05$)		Very low +	Important
**Long (2010)** [6]**, Tiwari (2010)** [8]**, Almeida (2021)** [7]**: EGCG effect on oxidative stress (follow-up of variable period)**											
**3**	Experimental rodent models of FASD.	No serious risk of bias	No serious inconsistency	Serious indirectness (different comparisons)	No serious imprecision	76	25	Oxidative stress improvement ($p < 0.05$)		Very low +	Important
**Tiwari (2010)** [8]**: EGCG and apoptosis prevention (28 days follow-up)**											
**1**	Experimental Wistar rat model of FASD.	No serious risk of bias	No serious inconsistency	No serious indirectness	No serious imprecision	28	7	Apoptosis reduction ($p < 0.05$)		Low ++	Important

**Long (2010)** [6]**, Almeida (2021)** [7]**: EGCG and growth restriction prevention (no follow-up)**										
**2**	Experimental C57BL/6 mouse models of FASD.	No serious risk of bias	No serious inconsistency	Serious indirectness (different comparisons)	No serious imprecision	48	18	Improvement in neural maturation and differentiation ($p < 0.05$)	Very low +	Important
**Tiwari (2010)** [8]**: EGCG prevention on cognitive impairment (28 days follow-up)**										
**1**	Experimental Wistar rat model of FASD.	No serious risk of bias	No serious inconsistency	No serious indirectness	No serious imprecision	28	7	Cognitive improvement ($p < 0.05$)	Low ++	Important

Abbreviations. EGCG: epigallocatechin-3-gallate. FASD: fetal alcohol spectrum disorders. Quality of evidence grades: low (++), very low (+).

Table S3. Summary of GRADE evidence profile for Down syndrome.

Quality Assessment					No. of Patients		Effect		Quality	Importance
**Nº of studies**	**Design**	**Risk of Bias**	**Inconsistency**	**Indirectness**	**Imprecision**	**Cases**	**Control**	**Relative (95%CI)**	**Absolute**		
**Gu et al., 2020** [9], **Catuara–Solarz et al., 2016** [10], **Valenti et al., 2016** [11], **Valenti et al., 2013** [12], **Souchet et al., 2015** [13], **De Toma et al., 2019** [14], **De Toma et al., 2020** [15], **Catuara et al., 2015** [16], **Stagni et al., 2016** [17]**: Ability of EGCG to restore intellectual disability (no follow-up)**											
**9**	Experimental Ts65Dn or overexpress DYRK1A mouse models of DS or cell cultures	No serious risk of bias	No serious inconsistency	No serious indirectness	No serious imprecision			EGCG treatment derived cognitive improvements linked to neuromodulatory effects at the hippocampus ($p < 0.05$)	-	Low ++	Important
**Goodlett et al., 2020** [18], **Stringer et al., 2015** [19], **Stringer et al., 2017** [20]**: Ability of EGCG to restore intellectual disability (no follow-up)**											
**3**	Experimental Ts65Dn mouse models of DS	No serious risk of bias	No serious inconsistency	No serious indirectness	No serious imprecision			EGCG was not found to produce beneficial therapeutic effects on behavior, learning, and memory ($p < 0.05$)	-	Low ++	Important
**de La Torre et al., 2014** [21], **de La Torre et al., 2016** [22]**: Ability of EGCG to restore intellectual disability (12 + six months of follow-up)**											
**2**	RDBPC	No serious risk of bias	No serious inconsistency	No serious indirectness	No serious imprecision	43 DS	41 controls	Participants treated with EGCG and cognitive training had significantly higher scores in memory, learning, and behavior subdomains ($p < 0.05$)	-	High ++++	Critical
**Starbuck et al., 2021** [23]**: Therapeutic potential of EGCG for ameliorating facial dysmorphologies associated with DS (follow-up of variable periods)**											
**1**	Case control study	Risk of bias for small n treated	No serious inconsistency	No serious indirectness	No serious imprecision	63 DS, four mosaics, 13 treated with EGCG	207 euploids	EGCG modulates facial development with dose-dependent effects ($p < 0.05$)	-	Moderate +++	Important

with EGCG

Abbreviations. EGCG: epigallocatechin,-3-gallate. DS: Down syndrome. RDBPC: randomized, double blind, placebo-controlled study. Quality of evidence grades: high (++++), moderate (+++), low (++).

Table S4. Summary of GRADE evidence profile on the neurologic effects of catechins in healthy populations.

Quality Assessment						No. of Patients		Effect		Quality	Importance
**Nº of Studies**	**Design**	**Risk of Bias**	**Inconsistency**	**Indirectness**	**Imprecision**		**Control**	**Relative (95% CI)**	**Absolute**		
**Kesse–Guyot et al., 2012** [24], **Biasibetti et al., 2013** [25]: **Association between polyphenol intake and cognitive function (13 years of follow-up)**											
2	Observational,	No serious risk of bias	No serious inconsistency	No serious indirectness	No serious imprecision	3382	-	Associations between quartiles of catechin intakes and language and verbal memory: Q1: 48.1 +/−0.7, Q2: 49.3 +/−0.7, Q3: 49.4 +/−0.7, Q4: 50.1 +/−0.7 p = 0.001; executive function: Q1: 51.3 +/−0.7, Q2: 51.0 +/−0.7, Q3: 50.1 +/−0.7, Q4: 49.9 +/−0.7, p = 0.01; cognitive status: Q4 vs. Q1: OR = 0.24, 95% CI: 0.08, 0.72	-	Low ++	Important
**Mohamed S, et al., (2013)** [26]: **Cognitive effects of OPLE (two months follow up)**											
1	RDBPC	No serious	No serious inconsistency	Serious indirectness	Serious imprecision	15	15	Short-term memory (p <	-	Low ++	Important

		risk of bias		(subrogated variables, selected population)	(low sample size)			0.001), spatial visualization ability and processing speed ($p < 0.05$) improved after of OPLE.			
**Scholey A, et al., (2012)**[27]**; Emma L, et al., (2012)** [28]**, Dietz C, et al.,(2017)**[29]**, Liu Y, et al., (2018)** [30]**: Effect of EGCG, green tea extract or matcha tea on brain and mood**											
4	RSBPC crossover trial	No serious	No Serious inconsistency	Serious indirectness (subrogated variables)	No serious imprecision	105	105	No differences on mood. Reduction in oxy-Hb only with 135 mg of EGCG ($p < 0.001$). EEG: increase in alpha, beta and theta activity with EGCG. Matcha: improvement in basic attention and psychomotor speed response. Decaffeinated GTE improved reading span only in older women ($p = 0.04$)	-	Moderate +++	Important
**Ide et al., (2016)** [31]**, Sakurai et al., (2020)** [32]**, Baba et al., (2020)** [33]**: Green tea intake on cognitive dysfunction (one year of follow-up)**											
3	RDBPC	No serious	No serious inconsistency	No serious indirectness	No serious imprecision	71	68	Global cognitive and memory	-	High ++++	Important

	risk of bias		function tests: no significant differences.	

Abbreviations: Q: quartile, OPLE: alcoholic oil palm leaves extract, EGCG: epigallocatechin-gallate, EEG: electroencephalogram, GTE: green tea extract, RDBPC: randomized, double blind, placebo-controlled study, RSBPC: randomized, single blind, placebo-controlled study. Quality of evidence grades: high (++++), moderate (+++), low (++).

References

1. Beasley M, Stonebraker AR, Hasan I, Kapp KL, Liang BJ, Agarwal G, et al. Lipid membranes influence the ability of small molecules to inhibit huntingtin fibrillization. Biochemistry. 2019;58(43):4361–73 . DOI: 10.1021/acs.biochem.9b00739.Lipid.
2. Varga J, Dér NP, Zsindely N, Bodai L. Green tea infusion alleviates neurodegeneration induced by mutant Huntingtin in Drosophila. Nutr Neurosci. 2020;23(3):183–9 . DOI: 10.1080/1028415X.2018.1484021. PMID: 29973113
3. Ehrnhoefer DE, Duennwald M, Markovic P, Wacker JL, Engemann S, Roark M, et al. Green tea (-)-epigallocatechin-gallate modulates early events in huntingtin misfolding and reduces toxicity in Huntington's disease models. Hum Mol Genet. 2006;15(18):2743–51 . DOI: 10.1093/hmg/ddl210. PMID: 16893904
4. Judith Bellmann-Strobl, Friedemann Paul, Jens Wuerfel, Jan D¨orr, Carmen Infante-Duarte, Elmira Heidrich, Benedict K¨ortgen, Alexander Brandt, Caspar Pf¨uller, Helena Radbruch, Rebekka Rust, Volker Siffrin, Orhan Aktas, Christoph Heesen, J¨urgen Faiss, Fr and FZ. Epigallocatechin Gallate in Relapsing-Remitting Multiple Sclerosis. A randomized, placebo-controlled trial. Neurol Neuroimmunol neuroinflammation. 2021;8(3) . DOI: 10.1212/NXI.0000000000000981.
5. Rust R, Chien C, Scheel M, Brandt AU, Dörr J, Wuerfel J, et al. Epigallocatechin Gallate in Progressive MS: A Randomized, Placebo-Controlled Trial. Neurol Neuroimmunol neuroinflammation. 2021;8(3) . DOI: 10.1212/NXI.0000000000000964. PMID: 33622766
6. Long L, Li Y, Wang YD, He QY, Li M, Cai XD, et al. The preventive effect of oral EGCG in a fetal alcohol spectrum disorder mouse model. Alcohol Clin Exp Res. 2010;34(11):1929–36 . DOI: 10.1111/j.1530-0277.2010.01282.x. PMID: 20659071
7. Almeida-Toledano L, Andreu-Fernández V, Aras-López R, García-Algar Ó, Martínez L, Gómez-Roig MD. Epigallocatechin gallate ameliorates the effects of prenatal alcohol exposure in a fetal alcohol spectrum disorder-like mouse model. Int J Mol Sci. 2021;22(2):1–24 . DOI: 10.3390/ijms22020715. PMID: 33450816
8. Tiwari V, Kuhad A, Chopra K. Epigallocatechin-3-gallate ameliorates alcohol-induced cognitive dysfunctions and apoptotic neurodegeneration in the developing rat brain. Int J Neuropsychopharmacol. 2010;13(8):1053–66 . DOI: 10.1017/S146114571000060X. PMID: 20529413
9. Gu Y, Moroy G, Paul JL, Rebillat AS, Dierssen M, de la Torre R, et al. Molecular rescue of DYRK1A overexpression alterations in mice with fontup® dietary supplement: Role of green tea catechins. Int J Mol Sci. 2020;21(4) . DOI: 10.3390/ijms21041404. PMID: 32092951
10. Catuara-Solarz S, Espinosa-Carrasco J, Erb I, Langohr K, Gonzalez JR, Notredame C, et al. Combined Treatment With Environmental Enrichment and (-)-Epigallocatechin-3-Gallate Ameliorates Learning Deficits and Hippocampal Alterations in a Mouse Model of Down Syndrome. eNeuro. 2016;3(5) . DOI: 10.1523/ENEURO.0103-16.2016. PMID: 27844057
11. Valenti D, de Bari L, de Rasmo D, Signorile A, Henrion-Caude A, Contestabile A, et al. The polyphenols resveratrol and epigallocatechin-3-gallate restore the severe impairment of mitochondria in hippocampal progenitor cells from a Down syndrome mouse model. Biochim Biophys Acta - Mol Basis Dis. 2016;1862(6):1093–104 . DOI: 10.1016/j.bbadis.2016.03.003.
12. Valenti D, De Rasmo D, Signorile A, Rossi L, de Bari L, Scala I, et al. Epigallocatechin-3-gallate prevents oxidative phosphorylation deficit and promotes mitochondrial biogenesis in human cells from subjects with Down's syndrome. Biochim Biophys Acta - Mol Basis Dis. 2013;1832(4):542–52 . DOI: 10.1016/j.bbadis.2012.12.011. PMID: 23291000
13. Souchet B, Guedj F, Penke-Verdier Z, Daubigney F, Duchon A, Herault Y, et al. Pharmacological correction of excitation/inhibition imbalance in down syndrome mouse models. Front Behav Neurosci. 2015;9(OCTOBER) . DOI: 10.3389/fnbeh.2015.00267.
14. De Toma I, Ortega M, Aloy P, Sabidó E, Dierssen M. DYRK1A Overexpression Alters Cognition and Neural-Related Proteomic Pathways in the Hippocampus That Are Rescued by Green Tea Extract and/or Environmental Enrichment. Front Mol Neurosci. 2019;12(November):1–22 . DOI: 10.3389/fnmol.2019.00272.
15. De Toma I, Ortega M, Catuara-Solarz S, Sierra C, Sabidó E, Dierssen M. Re-establishment of the epigenetic state and rescue of kinome deregulation in Ts65Dn mice upon treatment with green tea extract and environmental enrichment. Sci Rep. 2020;10(1) . DOI: 10.1038/s41598-020-72625-z. PMID: 32994493
16. Catuara-Solarz S, Espinosa-Carrasco J, Erb I, Langohr K, Notredame C, Gonzalez JR, et al. Principal component analysis of the effects of environmental enrichment and (-)-epigallocatechin-3-gallate on age-associated learning deficits in a mouse model of down

syndrome. Front Behav Neurosci. 2015;9(DEC):1–14 . DOI: 10.3389/fnbeh.2015.00330. PMID: 26696850

17. Stagni F, Giacomini A, Emili M, Trazzi S, Guidi S, Sassi M, et al. Short- and long-term effects of neonatal pharmacotherapy with epigallocatechin-3-gallate on hippocampal development in the Ts65Dn mouse model of Down syndrome. Neuroscience. 2016;333:277–301 . DOI: 10.1016/j.neuroscience.2016.07.031. PMID: 27457036

18. Goodlett CR, Stringer M, LaCombe J, Patel R, Wallace JM, Roper RJ. Evaluation of the therapeutic potential of Epigallocatechin-3-gallate (EGCG) via oral gavage in young adult Down syndrome mice. Sci Rep. 2020;10(1):1–17 . DOI: 10.1038/s41598-020-67133-z. PMID: 32591597

19. Stringer M, Abeysekera I, Dria KJ, Roper RJ, Goodlett CR. Low dose EGCG treatment beginning in adolescence does not improve cognitive impairment in a Down syndrome mouse model. Pharmacol Biochem Behav. 2015;138:70–9 . DOI: 10.1016/j.pbb.2015.09.002. PMID: 26363314

20. Stringer M, Abeysekera I, Thomas J, Lacombe J, Stewart RJ, Dria KJ, et al. Epigallocatechin-3-gallate (EGCG) consumption in the Ts65Dn model of Down syndrome fails to improve behavioral deficits and is detrimental to skeletal phenotypes. Physiol Behav. 2017;177:230–41 . DOI: 10.1016/j.physbeh.2017.05.003.Epigallocatechin-3-gallate.

21. De la Torre R, De Sola S, Pons M, Duchon A, de Lagran MM, Farré M, et al. Epigallocatechin-3-gallate, a DYRK1A inhibitor, rescues cognitive deficits in Down syndrome mouse models and in humans. Mol Nutr Food Res. 2014;58(2):278–88 . DOI: 10.1002/mnfr.201300325. PMID: 24039182

22. de la Torre R, de Sola S, Hernandez G, Farré M, Pujol J, Rodriguez J, et al. Safety and efficacy of cognitive training plus epigallocatechin-3-gallate in young adults with Down's syndrome (TESDAD): A double-blind, randomised, placebo-controlled, phase 2 trial. Lancet Neurol. 2016;15(8):801–10 . DOI: 10.1016/S1474-4422(16)30034-5. PMID: 27302362

23. Starbuck JM, Llambrich S, Gonzàlez R, Albaigès J, Sarlé A, Wouters J, et al. Green tea extracts containing epigallocatechin-3-gallate modulate facial development in Down syndrome. Sci Rep. 2021;11(1):1–13 . DOI: 10.1038/s41598-021-83757-1.

24. Kesse-Guyot E, Fezeu L, Andreeva VA, Touvier M, Scalbert A, Hercberg S, et al. Total and specific polyphenol intakes in midlife are associated with cognitive function measured 13 years later. J Nutr. 2012;142(1):76–83 . DOI: 10.3945/jn.111.144428. PMID: 22090468

25. Biasibetti R, Tramontina AC, Costa AP, Dutra MF, Quincozes-Santos A, Nardin P, et al. Green tea (-)epigallocatechin-3-gallate reverses oxidative stress and reduces acetylcholinesterase activity in a streptozotocin-induced model of dementia. Behav Brain Res. 2013;236(1):186–93 . DOI: 10.1016/j.bbr.2012.08.039. PMID: 22964138

26. Mohamed S, Lee Ming T, Jaffri JM. Cognitive enhancement and neuroprotection by catechin-rich oil palm leaf extract supplement. J Sci Food Agric. 2013;93(4):819–27 . DOI: 10.1002/jsfa.5802. PMID: 23001939

27. Scholey A, Downey LA, Ciorciari J, Pipingas A, Nolidin K, Finn M, et al. Acute neurocognitive effects of epigallocatechin gallate (EGCG). Appetite. 2012;58(2):767–70 . DOI: 10.1016/j.appet.2011.11.016. PMID: 22127270

28. Emma L. Wightman, Crystal F. Haskell, Joanne S. Forster RCV and DOK. Epigallocatechin gallate, cerebral blood flow parameters, cognitive performance and mood in healthy humans: a double-blind, placebo-controlled, crossover investigation. Hum Psychopharmacol. 2012;27:177–86 . DOI: 10.1002/hup. PMID: 7929019

29. Dietz C, Dekker M, Piqueras-Fiszman B. An intervention study on the effect of matcha tea, in drink and snack bar formats, on mood and cognitive performance. Food Res Int. 2017;99:72–83 . DOI: 10.1016/j.foodres.2017.05.002. PMID: 28784536

30. Liu Y, Fly AD, Wang Z, Klaunig JE. The Effects of Green Tea Extract on Working Memory in Healthy Women. J Nutr Heal Aging. 2018;22(3):446–50 . DOI: 10.1007/s12603-017-0962-8. PMID: 29484360

31. Ide K, Yamada H, Takuma N, Kawasaki Y, Harada S, Nakase J, et al. Effects of green tea consumption on cognitive dysfunction in an elderly population: A randomized placebo-controlled study. Nutr J. 2016;15(1):1–9 . DOI: 10.1186/s12937-016-0168-7. PMID: 27142448

32. Sakurai K, Shen C, Ezaki Y, Inamura N, Fukushima Y, Masuoka N, et al. Effects of matcha green tea powder on cognitive functions of community-dwelling elderly individuals. Nutrients. 2020;12(12):1–15 . DOI: 10.3390/nu12123639. PMID: 33256220

33. Yoshitake Baba, Shun Inagaki, Sae Nakagawa, Toshiyuki Kaneko MK and TT. Effect of Daily Intake of Green Tea Catechins on Cognitive Function in Middle-Aged and Older Subjects: A Randomized, Placebo-Controlled Study. Molecules. 2020;24(4265) . DOI:

10.3390/molecules25184265.
